# Data-driven global ocean modeling for seasonal to decadal prediction

**DOI:** 10.1126/sciadv.adu2488

**Published:** 2025-08-13

**Authors:** Zijie Guo, Pumeng Lyu, Fenghua Ling, Lei Bai, Jing-Jia Luo, Niklas Boers, Toshio Yamagata, Takeshi Izumo, Sophie Cravatte, Antonietta Capotondi, Wanli Ouyang

**Affiliations:** ^1^ School of Computer Science, Fudan University, Shanghai, China.; ^2^ Shanghai Artificial Intelligence Laboratory, Shanghai, China.; ^3^ Institute for Climate and Application Research (ICAR)/CPRM/CIC-FEMD/KLME/ILCEC, Nanjing University of Information Science and Technology, Nanjing, China.; ^4^ Earth System Modelling, School of Engineering and Design, Technical University of Munich, Munich, Germany.; ^5^ Potsdam Institute for Climate Impact Research, Potsdam, Germany.; ^6^ Application Laboratory, Japan Agency for Marine-Earth Science and Technology, Yokohama, Japan.; ^7^ Institut de Recherche pour le Développement (IRD), UMR241 SECOPOL (UPF/IRD/IFREMER/ILM) Laboratory, Université de la Polynésie Francaise (UPF), Tahiti, French Polynesia.; ^8^ LEGOS (IRD/UT3/CNES/CNRS), Université de Toulouse, Toulouse, France.; ^9^ French National Research Institute for Sustainable Development (IRD), Nouméa, New Caledonia.; ^10^ Cooperative Institute for Research in Environmental Sciences, University of Colorado Boulder, Boulder, CO, USA.; ^11^ NOAA Physical Sciences Laboratory, Boulder, CO, USA.; ^12^ Department of Information Engineering, The Chinese University of Hong Kong, Hong Kong, China.

## Abstract

Accurate modeling of ocean dynamics is crucial for enhancing our understanding of complex ocean circulation processes, predicting climate variability, and tackling challenges posed by climate change. Although great efforts have been made to improve traditional numerical models, predicting global ocean variability over multiyear scales remains challenging. Here, we propose ORCA-DL (Oceanic Reliable foreCAst via Deep Learning), a data-driven three-dimensional ocean model for seasonal to decadal prediction of global ocean dynamics. ORCA-DL accurately simulates the three-dimensional structure of global ocean dynamics with high physical consistency and outperforms state-of-the-art numerical models in capturing extreme events, including El Niño–Southern Oscillation and upper ocean heat waves. Moreover, ORCA-DL stably emulates ocean dynamics at decadal timescales, demonstrating its potential even for skillful decadal predictions and climate projections. Our results demonstrate the high potential of data-driven models for providing efficient and accurate global ocean modeling and prediction.

## Teaser

A breakthrough deep learning model is proposed for global three-dimensional ocean forecasting from seasonal to decadal scales.

## INTRODUCTION

Climate predictions from seasonal to decadal timescales are essential for society as they support resource management, planning, and long-term infrastructure investments in response to climate variability, and inform the design of climate change mitigation and adaptation strategies (*1*, *2*). Despite substantial international efforts over the past decade to enhance prediction skills (*3*), the accuracy of climate predictions is still limited. In addition, the reliability of climate projections is also often hampered by dynamical model deficiencies (*4*). Encouragingly, a body of research indicates that this challenge can be addressed by improving the modeling of more predictable subsystems within the climate system, particularly the ocean (*5*, *6*). As a primary heat reservoir, the ocean’s slowly evolving dynamics govern heat redistribution and are crucial for controlling weather patterns and climate change (*7*–*9*).

Traditionally, ocean general circulation models (OGCMs) have been used to simulate the time-evolving, three-dimensional (3D) ocean dynamics by numerically solving discretized forms of the Navier-Stokes equations (*10*). They can be coupled to atmospheric GCMs (AGCMs) to obtain coupled atmosphere–ocean general circulation models (AOGCMs) able to simulate and forecast climate and its modes of variability, such as the El Niño–Southern Oscillation (ENSO). Although these models have a robust physical framework, their accuracy is constrained by unavoidable trade-offs between spatial resolution and computational cost. To maintain tractability, subgrid-scale phenomena such as turbulent mixing and submesoscale eddies are approximated via parameterizations, which can introduce systemic biases (*10*, *11*). Furthermore, the slow development of dynamical models does not fully exploit the rapidly growing amount of satellite and in situ measurements, leading to increased uncertainty in the initializations and parameterizations of numerical models, further compromising the accuracy of predictions (*12*, *13*).

Although numerical AOGCMs grapple with resolution and parameterization challenges, statistical methods have served as a valuable complement. Empirical statistical models, such as linear regression, linear inverse modeling and principal components analysis, leverage historical observations or simulations to forecast key oceanic elements by capturing the statistical characteristics of climate subsystems (*14*–*17*). Dynamical-statistical hybrid frameworks enhance predictive capabilities by integrating simplified physical mechanisms with statistical methods (*18*, *19*). However, substantial limitations remain. The statistical models’ linear architectures prove inadequate for capturing nonlinear climate interactions, whereas hybrid frameworks may inadequately represent 3D dynamical processes due to dimensionality reduction and oversimplified conceptual equations. These persistent shortcomings underscore the need for more sophisticated nonlinear modeling paradigms. These challenges have driven efforts to develop more flexible nonlinear frameworks for ocean modeling.

Recently, there has been a surge in efforts to harness artificial intelligence (AI), particularly deep learning (DL), as a computationally efficient alternative to traditional methods. Unlike physical models that rely on explicit representations of fluid dynamics and empirical parameterizations, DL (or data-driven) models learn implicit relationships directly from observational or simulated data. Learning from atmospheric data, such models have achieved notable success in predicting global weather up to the medium range (*20*–*23*). DL models have also shown promising results in predicting specific climate indices [such as ENSO (*24*) or the Indian Ocean Dipole (*25*)] or regional ocean structures (*26*). Although these innovative DL approaches hold transformative potential, they are still far from achieving reliable simulations and predictions of the global 3D ocean.

Addressing these limitations requires overcoming many interconnected barriers. On the one hand, ocean data encompass millions of interacting grid points across multiple variables and depths. This high-dimensional nature requires models that can accurately capture 3D spatiotemporal dependencies while avoiding computational bottlenecks. Physical consistency is another key obstacle as models must preserve fundamental thermodynamic and dynamic relationships between variables to prevent unphysical drift in long-term predictions. This also requires unprecedented stability in the model’s autoregressive predictions to mitigate error accumulation. Last, the sparse subsurface ocean observations may prevent DL models from fully learning the complex relationships therein, further exacerbating these problems.

Here, to tackle these challenges, we present ORCA-DL (Oceanic Reliable foreCAst via Deep Learning), a data-driven model for global predictions of ocean dynamics from seasonal to decadal timescales. Inspired by traditional ocean modeling frameworks, ORCA-DL separately extracts signals of ocean variables and atmospheric forcing based on a powerful transformer architecture (*27*). A large set of simulations from the 6th phase of the Coupled Model Intercomparison Project (CMIP6) (*28*) are used for training to alleviate the problem of data scarcity and an effective multistep forecasting strategy is proposed to reduce the accumulated error. Through these designs, ORCA-DL achieves an accurate and efficient long-range forecast (see Materials and Methods for more information). Using reanalysis data over the past four decades as a predictand, ORCA-DL outperforms state-of-the-art dynamical models and demonstrates skillful predictions across three dimensions and multiple key variables, including salinity, potential temperature, and currents. ORCA-DL produces physically consistent forecasts for various climate phenomena, including ENSO and marine heat waves (MHWs), and can be stably run for decade-long simulations. These results highlight the ability of ORCA-DL to propel global ocean modeling, as well as to enhance the computational efficiency of climate predictions.

## RESULTS

ORCA-DL initializes with present monthly global oceanic states (including 3D temperature, salinity, and current fields) and atmospheric forcing mediated by zonal and meridional wind stress, generating monthly forecasts of the ocean (complete variable list and vertical discretization are shown in table S1). Our DL framework demonstrates autoregressive prediction capability analogous to dynamical models, enabling extended long-term prediction, and leveraging 10-member ensemble modeling to make more reliable predictions that account for inherent uncertainties. To evaluate the skill, data from the NCEP Global Ocean Data Assimilation System (GODAS) (*29*) are used as observational ground truth.

### Physical consistency of the mean state from ORCA-DL’s simulation

Given the persistent bias and climate drift in dynamical models, their simulated climate states are notably different from the real world (*30*, *31*). Therefore, evaluating the model’s climate state characteristics is crucial before assessing its skill in predicting climate variations, especially when using model-simulated data for training.

**Fig. 1.**
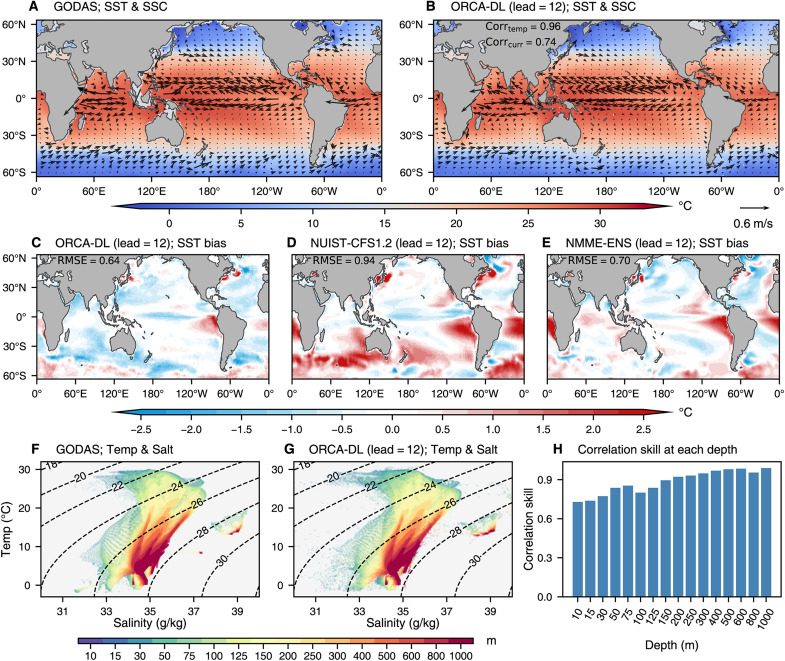
Climatological mean states in the DJF season (1985 to 2018). (**A** and **B**) Mean states of SST (shading) and SSC (vector) in the DJF season based on GODAS and ORCA-DL’s prediction at a lead time of 12 months. (**C** to **E**) Bias of SST mean states from ORCA-DL, NUIST-CFS1.2, and NMME-ENS relative to GODAS in the DJF season. NMME-ENS is the ensemble mean of seven models in the NMME. All models use a 12-month lead time forecast. (**F** and **G**) Scatterplots of the temperature and salinity mean states in the DJF season based on GODAS and ORCA-DL’s predictions at 12-month lead. The dashed contour lines denote potential density with a reference pressure of 0 dbar ( $\sigma_{0}$ density, kg/m^3^). The color of points represents depth. (**H**) Correlation for temperature-salinity distribution between ORCA-DL [as shown in (G)] and GODAS [as shown in (F)] at each depth.

Figure 1 shows the strong performance of ORCA-DL in simulating the climate mean states in December-January-February (DJF). It accurately simulates global sea surface temperature (SST) gradients and sea surface current (SSC) patterns, including the major ocean currents like the Antarctic Circumpolar Current, the Indian Ocean Gyre, and the North and South Equatorial Currents (Fig. 1, A and B). The skillful simulation of these currents is promising for accurately predicting climate phenomena that depend on ocean advection. Compared with traditional dynamical models like NUIST-CFS1.2 (*32*) and the North American Multi-Model Ensemble (*33*) ensemble mean of seven models (NMME-ENS) (see text S1.2.2), ORCA-DL substantially reduces the warm biases. Despite some underestimation of equatorial temperature, it maintains a lower root mean square error (RMSE) overall. We attribute ORCA-DL’s superior performance to the large amount of training data (a century-long simulation across dozens of models) that enables it to identify robust, physically consistent patterns. Although the CMIP6 models themselves are biased, our DL model can learn a complex nonlinear combination of all these models, thus generalizing beyond the limitations of any individual model (see text S3.3). Additional multiseasonal validations further support the model’s robustness (figs. S1 to S3).

Further evidence of physical consistency is provided by ORCA-DL’s simulation of temperature and salinity structures. Although there is a small divergence in the potential density (σ_0_) range of 24 to 26 kg/m^3^ in colder water, ORCA-DL has a highly consistent density distribution and maintains a high correlation coefficient with the observations at each depth (Fig. 1, F to H). Because the temperature and salinity values of water masses in different regions are different, we further visualized these characteristics for different water masses, which demonstrates that ORCA-DL can skillfully model the thermohaline properties at different depths and in different regions (figs. S4 and S5). Overall, these results demonstrate that ORCA-DL can accurately capture the 3D global oceanic states, in terms of density and currents, and the physical consistency among different variables.

### Skillful seasonal to interannual forecasting skills of ORCA-DL

#### Overall performance of ORCA-DL

**Fig. 2.**
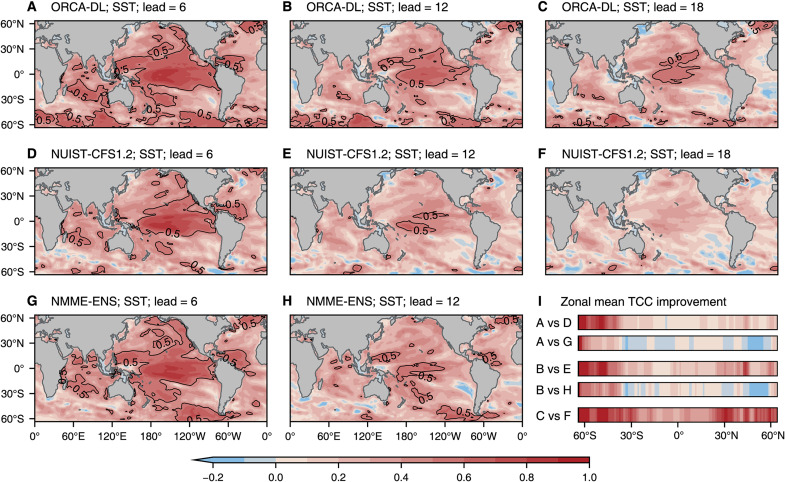
Distributions of TCC skills for SST anomaly prediction (1985 to 2018). (**A** to **C**) TCC skills of ORCA-DL for SST predictions at lead times of 6, 12, and 18 months, respectively. The black contour line is the dividing line where the prediction skill is equal to 0.5. (**D** to **F**) Skills for NUIST-CFS1.2. (**G** and **H**) Skills for NMME-ENS. (**I**) Relative improvement of zonal mean TCC skills of ORCA-DL compared to NUIST-CFS1.2 and NMME-ENS. The color bar is shared with (A) to (H). “A vs D” denotes the relative skill improvement of the 6-month lead prediction between ORCA-DL [as shown in (A)] and NUIST-CFS1.2 [as shown in (D)]. The others denote the corresponding skill difference.

To assess the prediction skill of ORCA-DL, we show the global geographical distribution of temporal correlation coefficient (TCC) skill in predicting SST anomalies for different models and lead times in Fig. 2. The highest skill appears in the tropics and especially in the tropical Pacific, both for ORCA-DL and other models. The SST anomalies in this region are the strongest and the most persistent and ENSO brings a relatively high predictability even at lead times up to 18 months (Fig. 2C). Compared with NUIST-CFS1.2, ORCA-DL shows superior skill across the entire time range. ORCA-DL is slightly worse in the high latitudes of the Northern Hemisphere and the mid-latitudes of the Southern Hemisphere when compared to NMME-ENS (Fig. 2I) but has better skill overall, especially at longer lead times (Fig. 2, B and H).

Beyond SST, we further assessed ORCA-DL’s performance across other ocean variables and depths. The spatial patterns of forecast skill are consistent with those for SST prediction, with particularly strong performance in the tropical Pacific and enhanced skill in the high latitudes of the Southern Hemisphere. These results are summarized in figs. S6 to S9, which show skill distributions of key ocean variables across different vertical layers and comparisons with the other models [NUIST-CFS1.2 and SINTEX-F (*34*)]. Notably, ORCA-DL can predict the deep ocean variations more accurately than the other models. However, this should not be interpreted as an inherent limitation of numerical modeling approaches. Instead, the performance gap primarily arises from the data assimilation challenges of traditional models. For example, NUIST-CFS1.2 demonstrates improved skill than SINTEX-F after adding the constraint of subsurface temperature observation in assimilation. This also indirectly proves that AI models can use observational data more quickly and economically.

In addition, the higher skills in the high latitudes of the Southern Hemisphere, which appear across multiple variables and depths, may be attributed to the greater variance of ocean variables in the extratropics at deeper layers (fig. S10). These larger natural fluctuations create complex patterns for the AI model to learn, which may lead the model to focus more on optimizing predictions for such regions during training (*35*), similar to the more accurate predictions of SST in the tropical Pacific region (Fig. 2, A to C).

#### Performance in predicting extreme climate phenomena

In addition to the overall prediction skills as reported above, we assess ORCA-DL’s capability to predict several key large-scale extreme climatic phenomena.

**Fig. 3.**
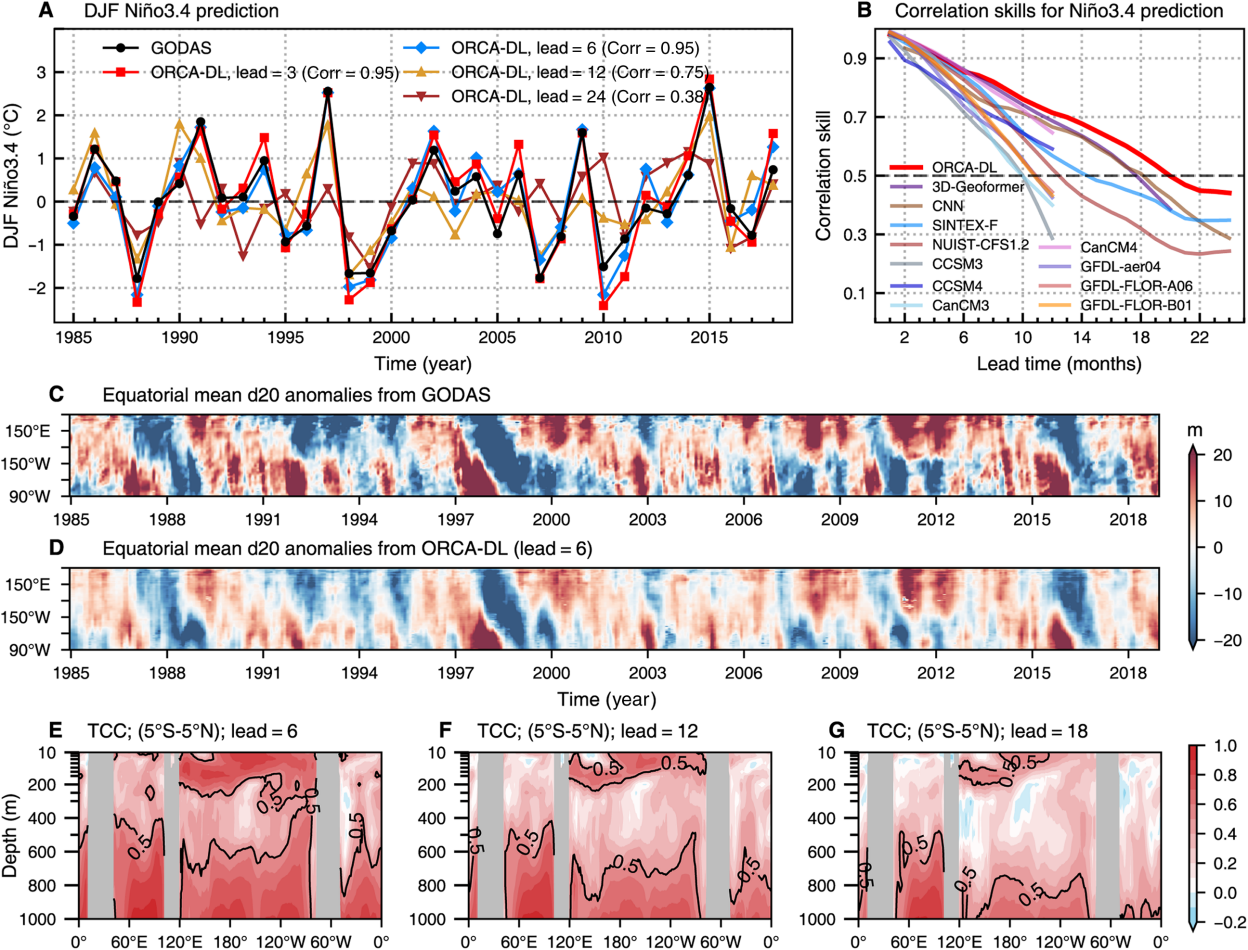
Skills of ORCA-DL in predicting ENSO. (**A**) DJF Niño3.4 index based on GODAS and predictions of ORCA-DL at lead times of 3, 6, 12, and 24 months, respectively. The *x* axis represents the winter season of each year, i.e., 2015 represents the period from December 2015 to February 2016. (**B**) Correlation skills of the Niño3.4 index prediction as a function of the lead month. The testing period is 1985 to 2018. (**C** and **D**) The 20°C isotherm depth (d20) anomalies averaged over latitude (5°S to 5°N) in the Pacific basin based on GODAS and ORCA-DL predictions at a lead time of 6 months, respectively. The *x* axis and *y* axis denote the time and longitude, respectively. (**E** to **G**) Depth-longitude distribution of TCC skills of temperature anomaly prediction in the equatorial region at lead times of 6, 12, and 18 months, respectively.

ENSO is one of the most prominent climate modes, centered in the central and eastern tropical Pacific Ocean. With global teleconnections, ENSO causes extreme atmospheric and oceanic anomalies worldwide (*36*, *37*). Predictive skill for this interannual mode has been pushed from seasonal to interannual timescales by DL approaches (*24*, *38*). As a crucial area for monitoring canonical ENSO behavior, the Niño3.4 region (170°W to 120°W, 5°S to 5°N) has been extensively used for ENSO studies (*39*, *40*). The Niño3.4 index during the DJF season reflects ENSO amplitudes during its peak phase. Hindcasts of the Niño3.4 index during the past four decades at different lead times indicate that ORCA-DL can correctly predict ENSO even at a long lead time (Fig. 3A). Although predicting ENSO becomes increasingly challenging with longer lead times, ORCA-DL has a high correlation skill of 0.75 at 12-month lead, overcoming the spring predictability barrier, and can still well predict the peaks of some extreme ENSO events, such as the La Niña events of 1988/1989 and 1999/2000 even up to 24-month lead and the 2015/2016 El Niño at 12-month lead.

To better assess the level of ORCA-DL’s skill for ENSO prediction, we compare it to the prediction skill of nine state-of-the-art process-based numerical models as well as two well-known data-driven models (Fig. 3B). ORCA-DL’s prediction skill for the Niño3.4 index surpasses that of not only the dynamical models but also the data-driven specific-task models [CNN (*24*) and 3D-Geoformer (*41*)] designed for ENSO prediction, especially for leads longer than 10 months, with a skillful prediction of the Niño3.4 index extending up to 20-month lead. In addition, ORCA-DL, similarly to ENSO-specific AI models, effectively captures the signal during ENSO phase transitions and is less affected by the spring predictability barrier (fig. S11).

Further analysis of the temporal evolution of the Niño3.4 area-averaged potential temperature anomalies, from the surface to a depth of 500 m, clarifies the reason why ORCA-DL achieves such accurate ENSO predictions (fig. S12). The subsurface anomalies exhibit an alternating pattern of warm and cold phases along the thermocline (i.e., the layer with the strongest vertical stratification), in association with the deepening and shallowing of the thermocline. They are related to equatorial Kelvin and Rossby waves propagating eastward and westward, respectively, which are crucial for the development and termination of El Niño and La Niña events (*42*), and seem to be partly captured by ORCA-DL, at least at 6- and 12-month lead times (Fig. 3, C and D, and fig. S13). These subsurface/thermocline signals often provide earlier indications of ENSO events and serve as a reliable source for prediction (*43*).

In addition, Fig. 3 (E to G) illustrates the depth-longitude distribution of TCC skills in predicting upper ocean temperature in the equatorial region, reinforcing ORCA-DL’s high performance. ORCA-DL demonstrates relatively high TCC skills at layers both above 200 m and below 500 m, particularly in the surface layer of the central Pacific, consistent with Fig. 2 (A to C). Conversely, traditional dynamical models encounter substantial forecasting difficulties at depths around 300 m, nearly losing predictive skill in deeper layers (fig. S12, F to I).

Similarly, as shown in fig. S14, ORCA-DL has also better skill than the other models in predicting the Central Pacific (CP) Niño4, and Eastern Pacific (EP) Niño3 indices, suggesting that ORCA-DL also better forecasts ENSO’s spatial diversity and flavors, as well as the related ENSO continuum between Warm-Pool El Niño, or Modoki events, and extreme Eastern Pacific El Niño events (*44*–*46*). ORCA-DL’s strong predictive performance extends beyond ENSO. It also shows skill in forecasting other important climate phenomena, such as the Indian Ocean Dipole and the Atlantic Niño.

MHWs are oceanic extreme events characterized by high ocean temperatures that can last from weeks to even years, with severe ecological and socioeconomic impacts (*47*–*49*). Although there is growing recognition of the need to improve upper ocean MHWs understanding and forecasts (*48*, *50*), most previous studies merely focused on surface MHWs due to limitations in ocean observations and numerical models’ performance (*51*, *52*). On the basis of the aforementioned assessments, we find that ORCA-DL exhibits superior multilayer forecasting capabilities. Consequently, we define upper ocean MHWs based on monthly ocean heat content above 300 m depth (*52*, *53*) and validate ORCA-DL’s capabilities for forecasting these MHWs. In simple terms, if the anomaly at a location exceeds the 90th percentile of anomalies for that location, it is considered that a heat wave event has occurred there (see text S2.2.1 for details).

**Fig. 4.**
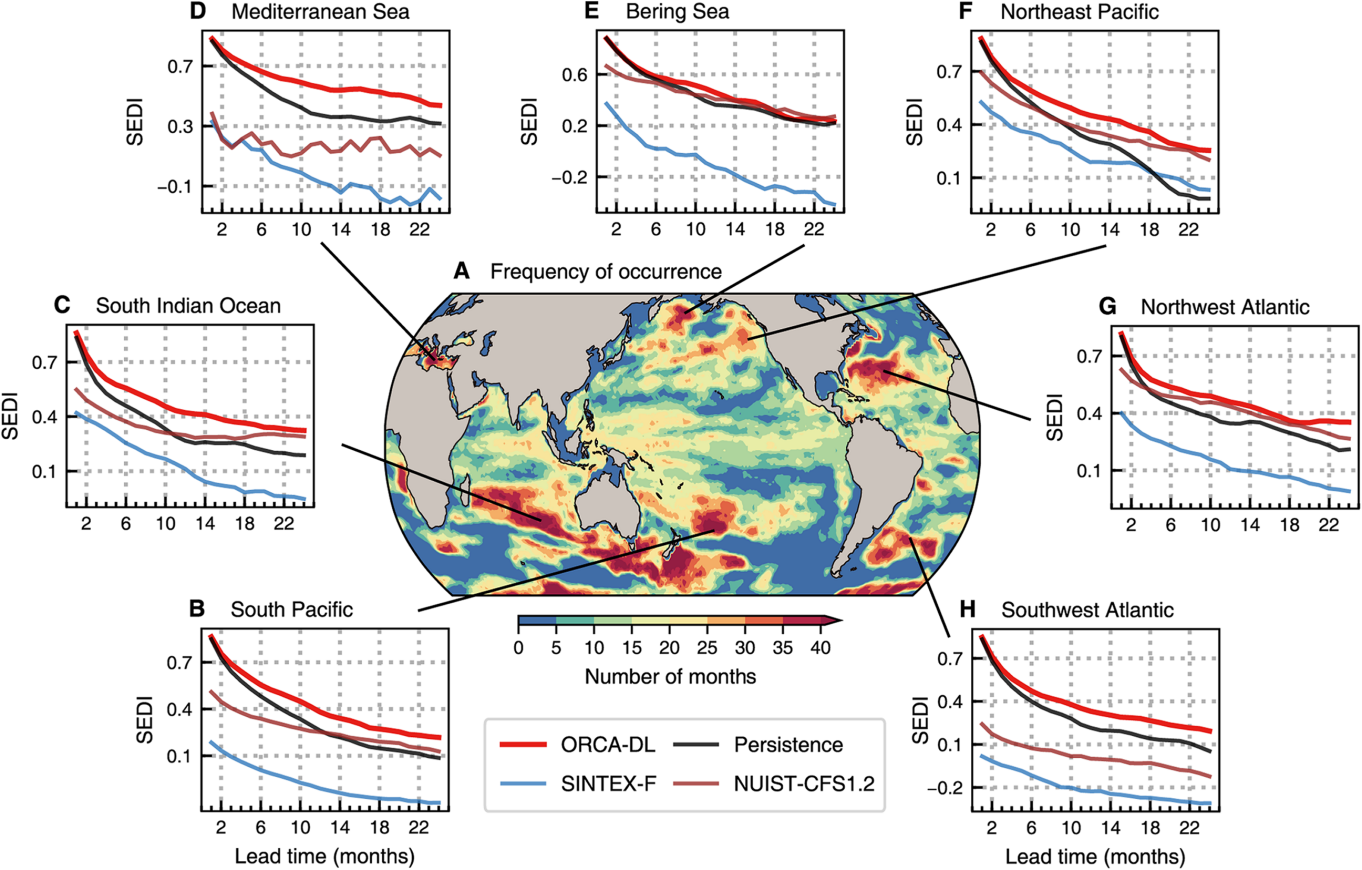
Forecast skills of upper ocean MWHs. (**A**) Distribution of the frequency of the occurrence of upper ocean MHW events based on GODAS from 2010 to 2018. (**B** to **H**) Regionally average SEDI (symmetric extremal dependence index) skill as a function of the lead time for seven selected regions with a relatively high frequency of heat wave occurrences. The skills are calculated based on MHW events between 1985 and 2018. The higher SEDI denotes the better skill.

Figure 4A shows the frequency distribution of upper ocean MHWs calculated using GODAS data during the period of 2010 to 2018 (note that the 90th percentile is calculated based on the period from 1985 to 2018). We select seven regions with a relatively high frequency of their occurrences to examine the predictive ability of ORCA-DL (see table S2 for specific locations). As shown in Fig. 4 (B to H), ORCA-DL performs exceptionally well in these areas, surpassing SINTEX-F, NUIST-CFS1.2, and persistence forecasts. It is noteworthy that traditional dynamical models struggle to exceed the accuracy of persistence forecasts. Compared with SINTEX-F, NUIST-CFS1.2 achieves comparable performance to ORCA-DL in some regions, possibly thanks to subsurface ocean data assimilation.

Regarding the forecast performance of surface MHWs, ORCA-DL either demonstrates a clear advantage or performs comparably well relative to all physical models (fig. S15). This further highlights ORCA-DL’s capability to accurately predict extreme events in both the surface and upper ocean.

### Potential of decadal prediction

Having explored the performance of ORCA-DL in seasonal and interannual forecasts, we extend our evaluation to decadal predictions, which provide a crucial foundation for informing adaptation strategies as the climate evolves (*54*). Although decadal predictions typically account for external influences, such as carbon dioxide emissions, our current focus is on assessing the model’s ability to produce stable long-term simulations and accurately reproduce climatology, seasonal cycles, and other variability modes internal to the climate system, including those occurring at decadal timescales.

**Fig. 5.**
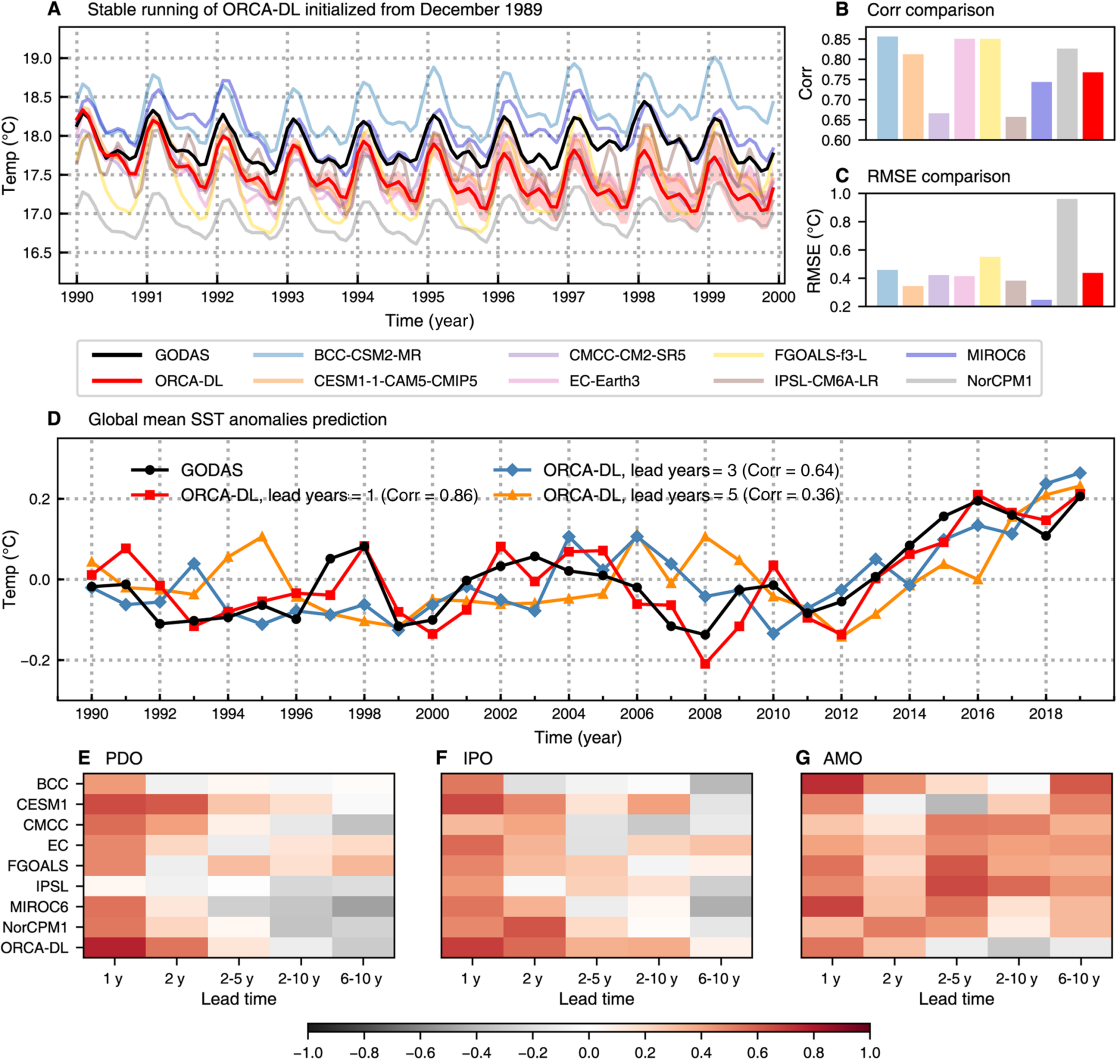
Decadal prediction of ORCA-DL. (**A**) Global mean SST based on GODAS, ORCA-DL, and eight dynamical models participated in the CMIP6 DCPP. ORCA-DL is initialized with field of December 1989. The red solid line indicates ORCA-DL’s ensemble mean results, whereas the red shadow indicates the spread of different members. (**B** and **C**) Comparison of correlation skills and RMSE between ORCA-DL and CMIP6 models in predicting the global mean SST shown in (A). (**D**) Global annual mean of SST anomalies based on GODAS and ORCA-DL’s predictions at lead times of 1, 3, and 5 years. The time range is selected as 1990 to 2019 to unify the forecasts for different lead times. (**E** to **G**) Correlation skills for the Pacific Decadal Oscillation (PDO), Interdecadal Pacific Oscillation (IPO), and Atlantic Multidecadal Oscillation (AMO) index forecast, respectively. The starting time of the test period is unified as 1990, and the end time of BCC, CESM1, FGOALS, and IPSL is 2014, 2018, 2017, and 2017, respectively, whereas the end time of other models is 2019. y, years.

Given that autoregressive forecasts over such long periods pose challenges for any dynamical and AI model due to the chaotic nature of ocean dynamics and correspondingly high error accumulation, we first verify that the model can run stably on the decadal timescale. Figure 5A illustrates a 10-year evolution of the global mean SST for GODAS, ORCA-DL, and eight dynamical models that participated in the CMIP6 Decadal Climate Prediction Project (DCPP) (*55*). Given the initial field in December 1989, ORCA-DL autoregressively predicts the ocean states from January 1990 to December 1999. As the lead month increases, ORCA-DL gradually exhibits a cold bias, which makes ORCA-DL lag behind several physical models in terms of correlation performance (Fig. 5B). These physical models are based on mature physical frameworks and are therefore usually stable in long-term simulations, thus having good correlation performance. However, most of them have higher RMSE than ORCA-DL due to inherent systematic biases partly caused by empirical parameterization (Fig. 5C).

Consistently, ORCA-DL produces similar results when initialized with fields from different years, exhibiting remarkable consistency with GODAS observations and underscoring its robust performance from different initial conditions (fig. S16). Typically, AI models based on autoregressive forecasting tend to accumulate errors quickly, leading to large biases. In contrast, our model maintains good stability in the decadal simulation. In addition, we note that ORCA-DL can still capture the seasonal cycles in SST without solar radiation as an input. One possible reason is that SSTs exhibit notable seasonal variations and ORCA-DL can implicitly learn the annual cycles during training by incorporating the lead time as input (see Materials and Methods).

However, as shown in fig. S17, we observed notable cold biases in the decadal mean-states of SSTs in some regions, such as the Kuroshio Extension region, the Atlantic Ocean, etc., which results in the colder global mean SST (Fig. 5A). In addition, the surface current velocity along the equator is notably underestimated. The cold (warm) biases in ORCA-DL without CO_2_ input are roughly consistent with the positive (negative) trends over the past 30 years in many regions (fig. S18), reflecting climate changes in response to the increased CO_2_ forcing. Such consistency is also preserved at deeper layers. This indicates the potential of ORCA-DL to enhance the reliability of decadal forecasts by incorporating realistic CO_2_ and other radiative forcing in the future.

To further explore the model’s ability to capture decadal variations, the prediction of global mean SST anomalies at different lead years is shown in Fig. 5D. As we can observe, although the correlation skills decrease at longer lead times beyond 5 years, it still effectively captures the overall trend. The skill for forecasting the indices of the Pacific Decadal Oscillation, Interdecadal Pacific Oscillation, and Atlantic Multidecadal Oscillation, as shown in Fig. 5 (E to G), further demonstrates that ORCA-DL exhibits excellent performance in capturing these important climate variations at a relatively short timescale of 1 to 2 years but also shows potential for decadal predictions.

## DISCUSSION

We presented ORCA-DL, a data-driven global ocean forecasting model for seasonal to decadal predictions. By training with historical simulations from 20 models in CMIP6, ORCA-DL exhibits the extraordinary forecast skill for almost all targeted variables. When compared to traditional AOGCMs, considerable improvements are seen in the tropical Pacific (e.g., ENSO) and in the Southern Ocean, with forecasting skill substantially improved at 12- to 24-month lead. The skillful predictions of ENSO and upper ocean MHWs demonstrate the ability of ORCA-DL to detect and predict key climate modes of variability and extreme events. The performance of ORCA-DL in capturing subsurface dynamics underscores its potential for accurately predicting and capturing subsurface variations. Furthermore, a decadal forecast of global SST with considerable correlation and RMSE skills demonstrates ORCA-DL’s long-term modeling capacities of the ocean. This result shows that ORCA-DL is a stable model with good preservation of ocean memory. Its capability of running stably over a decade also makes it a promising candidate for future climate projections.

Because of insufficient observational data at monthly resolution, we have to train ORCA-DL using the simulated data from a large ensemble of numerical climate models, which exhibit biases. Although the bias in the training data can be reduced by training on more diverse data, there is still ample room for further development toward more effective transfer learning approaches, tailored to the specific requirements of such a large model. In addition, we found that among the ocean variables modeled by the present model, multilayer variables play a key role in long-term predictions as the deep ocean has a stronger memory. See more details in texts S3.1 and S3.2.

It is also important to note that ORCA-DL’s ability to reproduce seasonal cycles without explicit radiative forcing inputs may reflect implicit dependencies on historical data statistics rather than physically grounded responses to external drivers. This design may limit its capacity to capture shifts in seasonal cycles (e.g., longer and hotter summers or shorter and milder winters) under changing radiative regimes. This means that the model’s predictions primarily reflect responses to different initial input fields, rather than accurately capturing the physical responses to changes in unmodeled variables (such as radiative forcing). Although ORCA-DL has been trained on hundreds of years of simulated climate variability, extrapolation beyond the training climate context should be approached with caution. Therefore, to achieve true decadal forecasts and address the challenge of nonstationary statistics under climate change, it is essential to incorporate anthropogenic climate indicators (e.g., CO_2_ concentrations, aerosol forcing, starting year, and so on) as time-varying inputs. Including these variables as dynamic inputs can allow the model to establish adaptive statistical relationships under evolving climate conditions.

Furthermore, ORCA-DL currently focuses only on ocean modeling because oceanic processes evolve more slowly, with notably much larger thermal inertia than the atmosphere. Thus, over long timescales, most of the climate system’s memory resides in the ocean, with here in our AI model the coupling with the atmosphere being implicitly taken into account (because the typical behavior of the ocean on which the AI model was trained includes the effects of ocean-atmosphere coupling). Following prior studies (*24*, *41*), we therefore adopt an ocean-only modeling strategy. However, stochastic high-frequency atmosphere signals, particularly in subtropical regions where ocean-atmosphere coupling is weak, play a nonnegligible role in driving the ocean. Thus, incorporating additional atmospheric variables (e.g., precipitation, surface air temperature, winds, and specific humidity) in the future could potentially enhance the reliability of climate predictions, similar to the traditional coupled dynamical models. Also, explicit incorporation of radiative forcing would enhance the physical realism of seasonal cycle projections, allowing ORCA-DL to directly simulate shifts in seasonal cycle and its intensity driven by external forcings. Another promising perspective of this ORCA-DL is that one could use it to perform sensitivity experiments with oceanic states being specified only in limited regions and see which one matters the most for prediction [as done in (*56*), in a much simpler model]. One could also use it to investigate how the long ocean memory is across various regions and depths. These sensitivity experiments would be computationally feasible, thanks to the cost-efficiency of running the model after it has been trained. Furthermore, we are particularly interested in incorporating physical constraints (e.g., conservation laws) into DL models such as ORCA-DL in our future studies.

## MATERIALS AND METHODS

### Datasets

The choice of modeling variables is critical to the ability to adequately represent the 3D ocean. For ocean state representation, ORCA-DL integrates SST and sea surface height (SSH) to account for surface heat and mass redistribution processes, whereas potential temperature and salinity at 16 layers (from the surface to 1000 m depth) represent water mass properties and thermohaline stratification. In addition, zonal and meridional current velocities at each layer are included to represent ocean circulation, energy transport, and wave dynamics. As we mentioned in Discussion, the ocean plays a dominant role in long-term forecasts, and part of low-frequency atmospheric signals may be implicitly captured by surface ocean variables, particularly in tropical regions where ocean-atmosphere coupling is strong. In contrast, the stochastic atmospheric variability in the subtropical regions is hardly unpredictable. Therefore, for the sake of simplicity, we do not incorporate a dedicated atmospheric module to explicitly forecast atmospheric states. Instead, we initialize the model with zonal and meridional wind stress and progressively modulate their influence on the ocean (see text S1.2.2 for details). This multivariate framework ensures that the model correctly captures ocean dynamical and thermodynamical processes from the surface to the deep layer. See table S1 for the complete modeled variables and depth levels.

The modeled variables are selected based on physical laws, but the performance of the model practically depends on the quality and quantity of the dataset used for training. Although observational records (e.g., GODAS) provide ground truth, their limited temporal coverage is insufficient for robustly training a 3D global model. We therefore used historical simulations from 20 models that participated in the CMIP6 for training (table S3). To help the trained model better emulate the real observation, the data before 1980 from reanalysis datasets—Simple Ocean Data Assimilation 2 (SODA2) (*57*) and Ocean Reanalysis System 5 (ORAS5) (*58*)—are used for model evaluation and parameter tuning. After the training, we conducted independent testing of ORCA-DL with GODAS reanalysis data from 1980 to 2019 (table S4).

For more easily modeling, all data mentioned above are interpolated into the regular grid (63.5°S to 63.5°N, 0.5°E to 359.5°E) with a resolution of 1° (128 × 360 grid points in total). Also, the multilevel variables are interpolated into predefined depths, including 16 layers in vertical (see table S1).

### ORCA-DL model

**Fig. 6.**
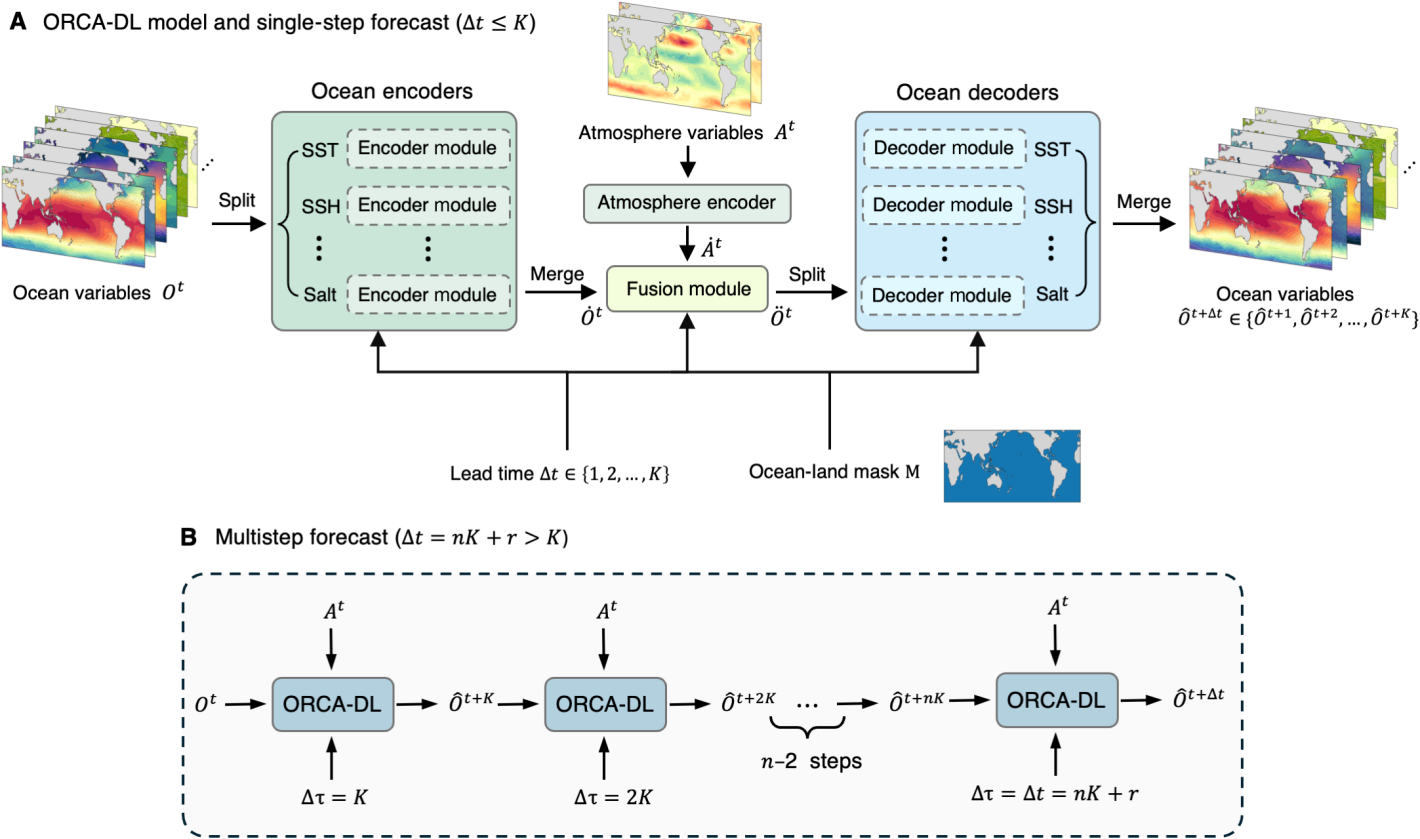
Architecture of the ORCA-DL model. (**A**) ORCA-DL consists of several ocean encoders, an atmosphere encoder, a fusion module, and several ocean decoders (skipping connections between each encoder module and decoder module are not shown for brevity). The inputs include present ocean and atmosphere variables $O^{t}$ and $A^{t}$ , ocean-land mask M , and lead time Δt . For a single-step forecast ( Δt≤K ), ORCA-DL outputs future ocean states ${\hat{O}}^{t+\Delta t}\in \left\{{\hat{O}}^{t+1},{\hat{O}}^{t+2},\ldots ,{\hat{O}}^{t+K}\right\}$ . K is the maximum interval that ORCA-DL can directly make a forecast (set to 6 in this study). (**B**) ORCA-DL performs a multistep forecast (ocean-land mask is not shown for brevity). Let Δt=nK+r(0≤r<K) , ORCA-DL rolls out n+1 steps ( n steps if r=0 ) to get the final prediction ${\hat{O}}^{t+\Delta t}$ . The first n steps use the interval of K and the last step uses r (if r>0 ). Δτ denotes the lead time of the intermediate prediction, i.e., the interval between output at each step and the initial field.

As illustrated in Fig. 6A, ORCA-DL mainly consists of several core components: ocean encoders ${ℰ}_{o}$ , atmosphere encoder ${ℰ}_{a}$ , fusion module ℱ, and ocean decoders 𝒟 (more detailed structures of each component are shown in fig. S19). These components work together to process input data, including ocean variables $O^{t}$ , atmospheric data (such as wind stresses) $A^{t}$ , ocean-land mask M , and the lead time Δt . The model is designed to generate future ocean states from these inputs and the workflow is shown in the following equations

$$\text{Encoded ocean states}\;{\dot{O}}^{t}={ℰ}_{o}\left(O^{t},M,\Delta t\right)$$ (1)

$$\text{Encoded atmosphere states}\;{\dot{A}}^{t}={ℰ}_{a}\left(A^{t},M,\Delta t\right)$$ (2)

$$\text{Fused states}\;{\ddot{O}}^{t}=ℱ\left({\dot{O}}^{t},{\dot{A}}^{t},\Delta t\right)$$ (3)

$$\text{Predicted ocean states}\;{\hat{O}}^{t+\Delta t}=𝒟\left({\ddot{O}}^{t},{\dot{O}}^{t},M,\Delta t\right)$$ (4)

Specifically, ORCA-DL first separately encodes each ocean variable in $O^{t}$ and atmosphere variables $A^{t}$ to obtain the high-dimensional signals of ocean and atmosphere variables ${\dot{O}}^{t}$ and ${\dot{A}}^{t}$ . Then, ${\dot{O}}^{t}$ is integrated in the fusion module with ${\dot{A}}^{t}$ to simulate the complex operations of the dynamical equations. Last, the decoders restore the calculated results to the spatial domain of each ocean variable, yielding next-step predictions. It should be noted that the model does not predict wind stress. The wind stress at the initial time is always used in the entire forecast process, and the impact of the initial field is controlled by the fusion module (fig. S19D).

The input $O^{t}$ and output ${\hat{O}}^{t+\Delta t}$ are high-dimensional matrices composed of all ocean fields at the time t and t+Δt , with a shape of $N_{o}\times N_{\text{lat}}\times N_{\text{lon}}=66\times 128\times 360$ . Similarly, $A^{t}$ contains wind stress in two directions and has a shape of $N_{a}\times N_{\text{lat}}\times N_{\text{lon}}=2\times 128\times 360$ . Ocean-land mask M has a shape of $N_{\text{lat}}\times N_{\text{lon}}=128\times 360$ and is used for boundary conditions, where the value is 0 in the ocean areas and 1 in the land areas. In a single-step forecast, the value range of lead time Δt is {1,2,..,K} months, where K is the maximum lead time that ORCA-DL can directly forecast. Because the model becomes more difficult to converge during training when K gets larger, we set K=6 in this study. To generate forecasts with a lead time longer than K months, we feed the model with the outputs ${\hat{O}}^{t+K}$ to get the next-step prediction. By repeating this operation multiple times, we can generate forecasts for any lead time, as shown in Fig. 6B.

### Training details

ORCA-DL is implemented with the Pytorch framework and trained with four Nvidia A100 GPUs within 12 hours using a total batch size of 32. The RMSE loss is used as the optimization target, which is defined in Eq. 5

$${ℒ}_{\text{RMSE}}=\sqrt{\frac{1}{N_{o}\times N_{\text{lat}}\times N_{\text{lon}}}\sum_{\nu ,i,j}\;{\left({\hat{O}}_{\nu ,i,j}^{t+\Delta t}-O_{\nu ,i,j}^{t+\Delta t}\right)}^{2}}\;$$ (5)

where ν,i,andj denote the index of variable, latitude, and longitude grid, respectively. We adopt the AdamW (*59*) as the optimizer using the following parameters: $\beta_{1}=0.9$ , $\beta_{2}=0.95$ , ϵ = 1 × 10^−6^ and $L_{2}$ weight decay = 0.1. The learning rate is warmed up with a ratio of 0.1 to a maximum value of 2 × 10^−4^, after which the cosine annealing is applied. Furthermore, to reduce the uncertainty introduced by autoregression while further decreasing error accumulation, 10 identical models are trained only with the difference of initial random seeds.

## Supplementary Materials

### This PDF file includes:

Supplementary Text Figs. S1 to S24 Tables S1 to S5 References
