# Supplementary material for: Data-driven global ocean modeling for seasonal to decadal prediction
Source: Sci Adv. 2025 Aug 13;11(33):eadu2488. doi: 10.1126/sciadv.adu2488 (PMC12346350; doi:10.1126/sciadv.adu2488)
Supplement: Supplementary file 1 — Supplementary Text Figs. S1 to S24 Tables S1 to S5 References [file sciadv.adu2488_sm.pdf]

Science Advances
AAAS

Supplementary Materials for

Data-driven global ocean modeling for seasonal to decadal prediction

Zijie Guo *et al.*

Corresponding author: Fenghua Ling, lingfenghua@pjlab.org.cn; Lei Bai, bailei@pjlab.org.cn;
Jing-Jia Luo, jjluo@nuist.edu.cn

Sci. Adv. **11**, eadu2488 (2025)
DOI: 10.1126/sciadv.adu2488

This PDF file includes:

Supplementary Text
Figs. S1 to S24
Tables S1 to S5
References

Supplementary Text

S1 Method Details

S1.1 Datasets

S1.2.1 Data Used by ORCA-DL

As mentioned in Materials and Methods, ORCA-DL is trained on historical simulations from 20 models that participated in the CMIP6 (Table S3). The time range is from 1850 to 2014, a total of 165 years, providing ORCA-DL with $165 \times 12 \times 20 = 39600$ months of samples. And because ORCA-DL can directly output predictions with lead time from 1 to $K = 6$ in single-step forecast and each lead time prediction corresponds to a label, the number of samples is further increased by 6 times. With such a large amount of data, ORCA-DL fully learn the complex ocean dynamics system.

To make the model more generalizable, the reanalysis data SODA2 and ORAS5 are used for validation every 200 steps of training. Specifically, the model with the lowest RMSE (best performance) on all validation samples in SODA2 and ORAS5 is retained for subsequent independent testing. Based on the performance of the model on the validation set, we can adjust the model and training parameters. The time periods used in SODA2 and ORAS5 are 1871-1979 and 1958-1979 respectively.

After the training phase, GODAS data from 1980 to 2019 is used for independent testing. The ORCA-DL in the testing phase is always initialized with fields from GODAS.

S1.2.2 Data Used for comparison

The North American Multi-Model Ensemble (NMME) is an experimental project, which was established in response to the U.S. National Academies' recommendation to support regional climate forecasting and decision-making over intra-seasonal to interannual timescales. The project has been contributing model predictions from their hindcasts (dating back to the early 1980s) and real-time forecasts since August 2011. Each model consists of 6–28 ensemble members, and the forecasts are provided at lead times from 1 month to 12 months (see https://iridl.ldeo.columbia.edu/SOURCES/.Models/.NMME/). Seven models are selected in this study: CCSM3, CCSM4, CanCM3, CanCM4, GFDL-aer04, GFDL-FLOR-A06, and GFDL-FLOR-B01.

The SINTEX-F prediction system is built based on a fully coupled global ocean-atmosphere circulation model developed under the EU-Japan collaborative framework. This forecast system with a coupled SST-nudging initialization scheme has displayed high performance in predicting the tropical climate signals (*34*). In particular, several ENSO events can be predicted at lead times of up to 2 years by this system. The real-time predictions have been updated every month and made publicly available since 2006 (see http://www.jamstec.go.jp/aplinfo/sintexf/e/seasonal/outlook.html).

The NUIST-CFS1.2 (Climate Forecast System of Nanjing University of Information Science and Technology) (*32*) is based on the fully coupled global ocean-atmosphere model SINTEX-F. This system employs an enhanced coupled SST-nudging scheme, utilizing the Ensemble Kalman Filter (EnKF) to assimilate multi-layer ocean data at the end of each month. The assimilated data encompasses full-field assimilation of observed sea surface temperature (SST), in-situ temperature and salinity profiles, as well as the assimilation of satellite-observed sea level anomalies (SLA). This approach enhances forecasting skills, particularly for subsurface condition, compared to the original SINTEX-F model (see https://icar.nuist.edu.cn/en/main.psp).

DCPP (Decadal Climate Prediction Project), a core component of CMIP6, aims to enhance climate prediction capabilities on interannual to decadal timescales. It comprises three modules: Component A builds a prediction database through multi-model historical hindcasts, evaluates model skill, and optimizes initialization methods; Component B conducts quasi-real-time multi-model predictions to explore potential operational forecasting; Component C investigates mechanisms of key climate events by analyzing interactions between natural variability and external forcings (see https://gmd.copernicus.org/articles/9/3751/2016/). Eight models from Component A are used: BCC-CSM2-MR, CESM1-1-CAM5-CMIP5, CMCC-CM2-SR5, EC-Earth3, FGOALS-f3-L, IPSL-CM6A-LR, MIROC6 and NorCPM1. Only one member (r1i1p1f1) of each model is used.

S1.2 Model

Inspired by traditional ocean modeling frameworks, ORCA-DL's initial condition consists of multiple layers of ocean variables—temperature, salinity, and currents (see Table S1 for complete modeling variables), while incorporating coastal lines as boundary conditions. Driving forces like wind stress on the ocean surface also influence ocean changes and are thus included. Since this work focuses solely on the upper 1000 meters of the ocean, bottom boundary conditions are not considered. In the modeling process, ORCA-DL independently encodes different variables to extract their own high-dimensional information. This information is then integrated in the fusion module to simulate the complex ocean dynamics. Finally, the decoder restores the calculated results to the spatial domain of each ocean variable, yielding next-step predictions. The overall architecture of ORCA-DL is illustrated in Fig. 6A. The components in ORCA-DL are introduced one by one below (also illustrated in Fig. S19).

S1.2.1 Ocean Encoders and Decoders

The ocean encoders and decoders consist of several ocean variable specific encoder and decoder modules respectively. As ORCA-DL separately encodes and decodes each ocean variable, each variable corresponds to an encoder and decoder module. For each variable, the encoder module first patchifies it with a patch embedding layer, which is a common operation in computer vision (*60*). Several consecutive local attention blocks then extract the high-level information. To capture features at different spatial scales while saving memory, down-sampling is conducted after every attention block except the last one following the implementation in Swin Transformer (*61*). The decoder module has an inverse process like the encoder.

S1.2.2 Fusion Module

Fusion Module takes the merged ocean hidden states encoded by all encoder modules, atmosphere hidden states encoded by the atmosphere encoder, and the lead time Δt as inputs. Inspired by the commonly used Rotary Position Embedding (RoPE) (*62*) in natural language processing, we transfer the position encoding to time encoding and propose the Time Rotary Fusion module.

Let $\boldsymbol{x}_{\mathrm{o}}, \boldsymbol{x}_{\mathrm{a}} \in \mathbb{R}^d$ denotes the hidden states vector of a single grid in ocean and atmosphere hidden states respectively, where d is hidden dims. We can get the rotated atmosphere vector $\boldsymbol{x}'_{\mathrm{a}} = \boldsymbol{R}^d_{\Theta,\Delta t}\boldsymbol{x}_{\mathrm{a}}$, where $\boldsymbol{R}^d_{\Theta,\Delta t}$ is the rotary matrix with pre-defined parameters $\Theta = \{\theta_i = 10000^{-2(i-1)/d}, i \in [1, 2, \ldots, d/2]$ defined as follows:

$$\boldsymbol{R}^d_{\Theta,\Delta t} = \begin{pmatrix} \cos\theta_1\Delta t & -\sin\theta_1\Delta t & 0 & 0 & \cdots & 0 & 0 \\ \sin\theta_1\Delta t & \cos\theta_1\Delta t & 0 & 0 & \cdots & 0 & 0 \\ 0 & 0 & \cos\theta_2\Delta t & -\sin\theta_2\Delta t & \cdots & 0 & 0 \\ 0 & 0 & \sin\theta_2\Delta t & \cos\theta_2\Delta t & \cdots & 0 & 0 \\ \vdots & \vdots & \vdots & \vdots & \ddots & \vdots & \vdots \\ 0 & 0 & 0 & 0 & \cdots & \cos\theta_{\frac{d}{2}}\Delta t & -\sin\theta_{\frac{d}{2}}\Delta t \\ 0 & 0 & 0 & 0 & \cdots & \sin\theta_{\frac{d}{2}}\Delta t & \cos\theta_{\frac{d}{2}}\Delta t \end{pmatrix} \tag{S1}$$

Then, the fused hidden vector $\boldsymbol{x}_{\mathrm{f}}$ can be calculated as follows:

$$\boldsymbol{x}_{\mathrm{f}} = \boldsymbol{x}_{\mathrm{o}} + \langle \boldsymbol{x}_{\mathrm{o}}, \boldsymbol{x}'_{\mathrm{a}} \rangle \cdot \boldsymbol{x}_{\mathrm{a}} \tag{S2}$$

Where $\langle\cdot\rangle$ denotes the inner product. When Δt gets larger, the relation between $\boldsymbol{x}_{\mathrm{o}}$ and $\boldsymbol{x}_{\mathrm{a}}$ gets weaker, quantified by $\langle \boldsymbol{x}_{\mathrm{o}}, \boldsymbol{x}'_{\mathrm{a}} \rangle$ in Eq. S2 (see also the original paper (*62*) for a detailed theoretical demonstration). With the Time Rotary Fusion module, we can fuse the present input ocean hidden states and the initial atmosphere hidden states (present and initial conditions can be at a different time when doing autoregressive forecast), which avoids the challenging forecast of the wind stress (*41*) and improves ORCA-DL's ability to manage the impact of atmospheric forcing on the ocean.

In order to further facilitate the model in extracting global information, position embedding (*60*) and several consecutive global attention blocks are added after fusion.

S1.2.3 Attention Block

Attention block is mainly based on Swin Transformer. It contains two successive sub-blocks. The first sub-block employs window-based multi-head self-attention (W-MSA) to capture the local information, while the subsequent sub-block uses shifted W-MSA (SW-MSA) for interactions across the windows. We introduce the ocean-land mask M to force the model to focus only on the ocean realm, similar to XiHe (*63*). To reduce the forecast steps and effectively simulate temporal differentiation or integration, we replace the single MultiLayer Perceptron (MLP) module with a group of MLPs like the prevailing module Mixture-of-Experts (MoE) (*64*) used in natural language processing and use the lead time Δt to index which MLP to use. This approach not only alleviates the accumulation of errors but also allows the model to select different precursors at varying lead times, enhancing its capacity to capture seasonal cycles and effective simulation of temporal differentiation or integration, just like the basic temporal ordinary differential equations (ODE). The overall attention block is computed as follows:

$$
\begin{aligned}
\hat{Z}^{l} &= \mathrm{W-MSA}(\mathrm{LN}(Z^{l-1}),\mathrm{M}) + Z^{l-1},\\
Z^{l} &= \mathrm{MLP}_{\Delta t}\left(\mathrm{LN}(\hat{Z}^{l})\right) + \hat{Z}^{l},\\
\hat{Z}^{l+1} &= \mathrm{SW-MSA}(\mathrm{LN}(Z^{l}),\mathrm{M}) + Z^{l},\\
Z^{l+1} &= \mathrm{MLP}_{\Delta t}\left(\mathrm{LN}(\hat{Z}^{l+1})\right) + \hat{Z}^{l+1}
\end{aligned}
\tag{S3}
$$

where LN denotes the Layer Normalization (*65*), M is the ocean-land mask, $\hat{Z}^l$ and Z^l represent the output features of the (S)W-MSA and MLP for block l, respectively. The attention used inside the (S)W-MSA is defined as follows:

$$
\mathrm{Attention}(Q,K,V) = \mathrm{SoftMax}\left(\frac{QK^T}{\sqrt{d}} + \mathrm{M} * (-100)\right)V \tag{S4}
$$

where Q, K, and V represent the query, key, and values vectors, and d is the hidden dimensions. Instead of using the relative position bias (*61*), we employ the RoPE to implicitly learn the relative position information. Moreover, the ocean-land mask M is added to the attention matrix to mask the land realm.

S1.2.4 Atmosphere Encoder

Atmosphere encoder shares the same structure with the ocean encoder module but only uses one MLP in the attention sub-block as we do not forecast the atmosphere variables but employ the Fusion Module as mentioned above.

S2. Evaluation Metrics and Definitions

S2.1 Evaluation Metrics

S2.1.1 RMSE

The Root Mean Square Error (RMSE) is a commonly used metric for evaluating how close a prediction is to the observation. Given the predicted values $\hat{O}^{t+\Delta t}$ and the observed values $O^{t+\Delta t}$, the RMSE of the prediction on a specific grid can be calculated as follows:

$$
\mathrm{RMSE}(v,i,j,\Delta t) = \sqrt{\frac{1}{T}\sum_{t}\left(\hat{O}_{v,i,j}^{t+\Delta t} - O_{v,i,j}^{t+\Delta t}\right)^2} \tag{S5}
$$

Where v denotes the specific variable or layer of multi-level variables, and T is the number of test time points. To obtain the RMSE value for a region, we simply average the RMSE of each grid within the region.

S2.1.2 TCC

The Temporal Correlation Coefficient (TCC) is a statistical measure used to evaluate how well a model is able to capture and reproduce the temporal phases of observed anomalies. The TCC of the prediction on a specific grid can be computed as follows:

$$\mathrm{TCC}(v,i,j,\Delta t) = \frac{\sum_t \left(\hat{O}_{v,i,j}^{t+\Delta t} - \hat{C}_{v,i,j}^{\mathrm{m}_{t+\Delta t}}\right)\left(O_{v,i,j}^{t+\Delta t} - C_{v,i,j}^{\mathrm{m}_{t+\Delta t}}\right)}{\sqrt{\sum_t \left(\hat{O}_{v,i,j}^{t+\Delta t} - \hat{C}_{v,i,j}^{\mathrm{m}_{t+\Delta t}}\right)^2 \sum_t \left(O_{v,i,j}^{t+\Delta t} - C_{v,i,j}^{\mathrm{m}_{t+\Delta t}}\right)^2}} \quad (S6)$$

Where C and $\hat{C}$ denote the forecast and observed climatology, $\mathrm{m}_{t+\Delta t}$ denotes the month corresponding to the time $t + \Delta t$. Additionally, the anomalies are not detrended unless decadal predicted skill, and the correlation skills all refer to the Pearson correlation coefficient.

S2.1.3 SEDI

The Symmetric Extremal Dependence Index (SEDI) (*66*) is a measure for rare binary event forecast with several advantages, including non-degenerate, base-rate independent, asymptotically equitable, and so on. In this study, we use SEDI to assess the forecast performance of subsurface marine heatwaves. It is defined as follows:

$$\mathrm{SEDI} = \frac{\log F - \log H - \log(1-F) + \log(1-H)}{\log F + \log H + \log(1-F) + \log(1-H)} \quad (S7)$$

where H is the hit rate (true positive rate) and F is the false alarm rate (false positive rate). SEDI has a range from -1 to 1, and a higher value indicates better performance.

S2.2 Definitions

S2.2.1 Definition of Upper Ocean MHWs

In this study, upper ocean MHWs are defined with ocean heat content (OHC) following that of (*53*). The OHC can be calculated as follows:

$$\mathrm{OHC} = c_p \rho \int_{z_1=0\mathrm{m}}^{z_2=300\mathrm{m}} T(z)dz \quad (S8)$$

where c_p is the specific heat capacity of seawater (3996 J/(kg.C)), ρ is the density (1026 kg/m^3), and $T(z)$ is the potential temperature at the depth z. The OHC anomalies are calculated first, then the MHW threshold corresponding to each month is calculated as the 90th percentile anomalies in a 3-month sliding window. For example, the threshold for January is the 90th percentile of all December to February OHC anomalies. Then, an MHW event is identified when the anomalies are greater than the 90th percentile of the corresponding month (*52*, *67*, *68*). The climatology and 90th percentile are calculated based on the period of 1985-2018.

S2.2.2 Calculation of Decadal Index

Pacific Decadal Oscillation (PDO) is defined as the EOF's first mode of SST anomalies in the North Pacific Basin (north of 20°N). The SST anomaly is obtained by removing the corresponding climatology from the SST at each grid point and then removing the long-term trend of the climate.

Interdecadal Pacific Oscillation (IPO) is represented by the TPI index (*69*). Three regions are defined first: (25°N-45°N, 140°E-145°W), (10°S-10°N, 170°E-90°W), and (50°S-15°S, 150°E-160°W). Then calculate the averaged SST anomalies in the three regions and get SSTA1, SSTA2, SSTA3. Finally, TPI = SSTA2 − (SSTA1 + SSTA3)/2.

Atlantic Multidecadal Oscillation (AMO) is calculated as the averaged SST anomalies in the North Atlantic (north of 0°) and the time series is detrended.

S3 Additional Results

S3.1 Contribution of Training Data

In order to explore the impact of training data on model performance, we first considered the CMIP6 models from three aspects: surface temperature biases, ENSO phase-locking biases, and marine heat waves (MHWs) prediction ability according to references (*70-72*), and divided the 20 CMIP6 models used for training into two groups; one with relatively high quality (top 10) and the other with relatively low quality as shown in Table S5. Afterwards, we retrained two ORCA-DL models using these two sets of input data. Since the number of training samples was reduced by half, we trained them twice as many steps to align with the original model. As shown in Fig. 20, we compared the performance of the two models with the original model in Niño3.4 index prediction and upper ocean MHWs prediction. It can be seen that the model trained with high-quality data performs better than that trained with low-quality data, but the former is still worse than that trained with all 20 CMIP6 models (i.e., the original model). This demonstrates that low-quality data is not without effect, and it also contributes to the learning during model training. Therefore, we believe that using more diverse data can achieve better performance.

S3.2 Contribution of Input Variables

To explore the impact of different variables on model performance, we conducted a series of sensitivity experiments. In the first experiment, we replaced different variables in the input of ORCA-DL with their climatological mean states, keeping the others unchanged. Fig. S21 demonstrates the differential impacts of variable substitution on ENSO prediction and MHW forecasting.

For ENSO prediction, the performance decreases the most after replacing the SST initial condition with its climatology, which is in line with the fact that the Niño index is calculated based on the SST anomalies. The result shows that the Nino3.4 prediction skill drops sharply at short lead times and then decreases gradually with the increasing lead time. The reason is that at a lead time of 1 month, the ENSO event may have occurred, so the important information of the initial SST is lost after replacing with its climate state, resulting in a significant decrease in the performance. Then the model skill rebounds because other variables (such as subsurface temperature and currents) are correctly predicted. Although the performance without initial SST decreases a lot, it still maintains

a certain skill, and after a lead time of 18 months, the performance is comparable to the original model. If replacing initial salinity, potential temperature and currents with their climatology, the performance also becomes lower but without the rapid drop at short lead times. The results suggest that missing one variable may always degrade the model performance albeit with different impacts of different variables.

For upper ocean MHWs prediction, initial potential temperature plays the most important role, because upper ocean MHWs are defined based on ocean heat content (see section S2.2.1). The impact of other variables is relatively small, possibly because of the strong memory of ocean heat content and missing one of the other variables does not appear to affect much the prediction of the evolution of heat content. Note that the slightly changed threshold of the 90th percentile of the predicted heat content among the different sensitivity experiments may also impact the skill.

To further explore the impact of the initial conditions on the model performance, we keep only one variable unchanged but replace all other variables with their climatology and conduct another set of sensitivity experiments. As illustrated in Fig. S22A, the importance of initial SST rapidly decreases after several months while the importance of subsurface temperature and ocean current dominates at lead times of beyond 6 months. This is also consistent with the common notion that the memory of subsurface ocean helps keep the long-range predictability of ENSO. In contrast, initial salinity does not seem to play a vital role in the mid-long lead forecast of ENSO. For marine heatwave (MHW) prediction (Fig. S22B), initial potential temperature plays a dominant role with the second and third contribution from SST and ocean current. Again, initial salinity plays the least role. Since the MHWs are defined based on temperature anomalies in upper ocean (typically 0–300m), these results fully proves the necessity of multi-layer modeling.

S3.3 Bias Comparison with CMIP6

The surface SST biases shown in Fig. 1C reflect the well-known cold equatorial and warm east boundary current biases reported in CMIP6 models. Therefore, we compared the SST mean state bias between ORCA-DL forecast with a lead time of 12 months and CMIP6 ensemble mean (CMIP6-ENS) as shown in Fig. S23. It can be observed that ORCA-DL significantly reduces the warm bias in the CMIP6 models and maintains a lower cold bias overall. An interesting question is why the bias of CMIP6 is larger than the bias of ORCA-DL trained on CMIP6. We believe that one possible reason is that our deep learning (DL) framework leverages the collective diversity of multiple CMIP6 models, rather than relying on the individual model. While single CMIP6 simulation may exhibit biases, aggregating across models provides a rich dataset that statistically samples a broader range of variability and uncertainties. The vast volume of training data (century-long simulations across dozens of models) allows the DL model to identify robust, physically consistent patterns and generalize beyond the limitations of any single model. This is also demonstrated by Fig. S20, where the DL model trained on all the CMIP6 model data performs better than the DL model trained on part of the CMIP6 model data, even when trained on a higher quality subset of CMIP6 models.

S3.4 Rollout with Evolving Wind Stresses

Although the atmosphere is also important, the ocean plays a dominant role in long-term forecasts due to its strong memory, as was demonstrated by the CNN DL model of Ham et al. (*24*) that only

used upper ocean heat content and SST as input for skillful 17-month ENSO forecast. However, in the ORCA-DL model, there is a lack of a module for modelling the effect of the ocean on the atmosphere; we think the rolling forecast of wind stress is unreasonable, so we designed the Fusion Module to control the impact of the initial wind stress on the ocean forecast. Since this control is based on the lead time (Fig. S19D), we can also input instantaneous wind stress. We only need to change the lead time input into the Fusion Module to the difference between the target forecast time and the time of the input instantaneous wind stress, rather than the difference between the target forecast time and the initial field.

To further verify the impact of the instantaneous wind stress input on the ENSO prediction performance, we conducted corresponding experiments. As shown in Fig. S24, the forecasts with the instantaneous wind stress input perform comparable to the original forecasts, except for slightly worse performance at lead time of 6 to 18 months. We believe the reason for the performance degradation is that the model uses the initial wind stress instead of the instantaneous wind stress during training. The corresponding degrees of decay are different, so there is a minor gap in the final performance.

S4 Supplementary Figures and Tables

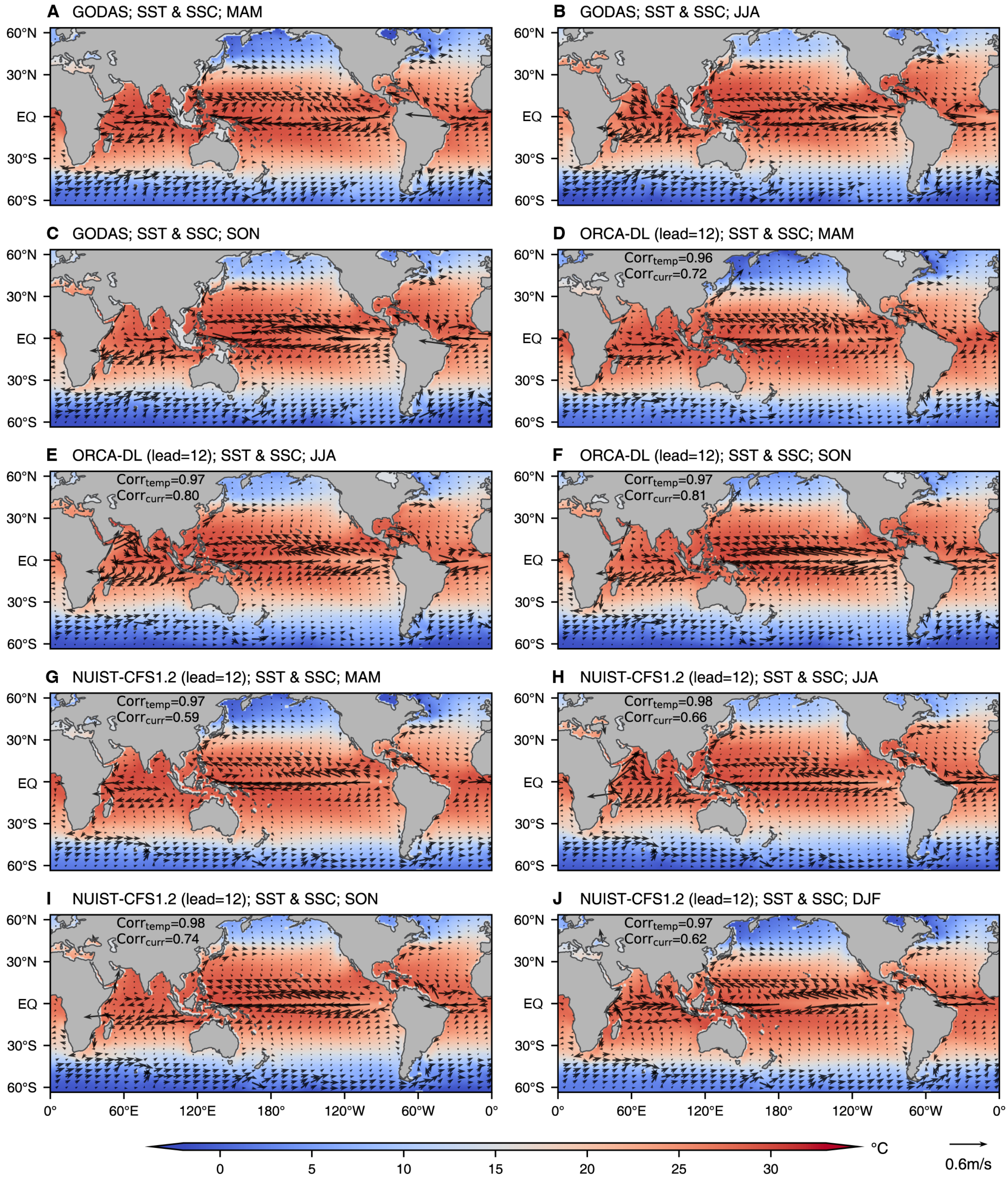

Fig. S1. Climatological mean state of SST and SSC based on the period of 1985-2018. (**A** to **C**) The mean state in MAM, JJA, SON seasons based on GODAS. (**D** to **F**) As in (A) to (C), but for ORCA-DL's prediction at a lead time of 12 months. (**G** to **J**) As in (A) to (C), but for NUIST-CFS1.2 forecast in four seasons.

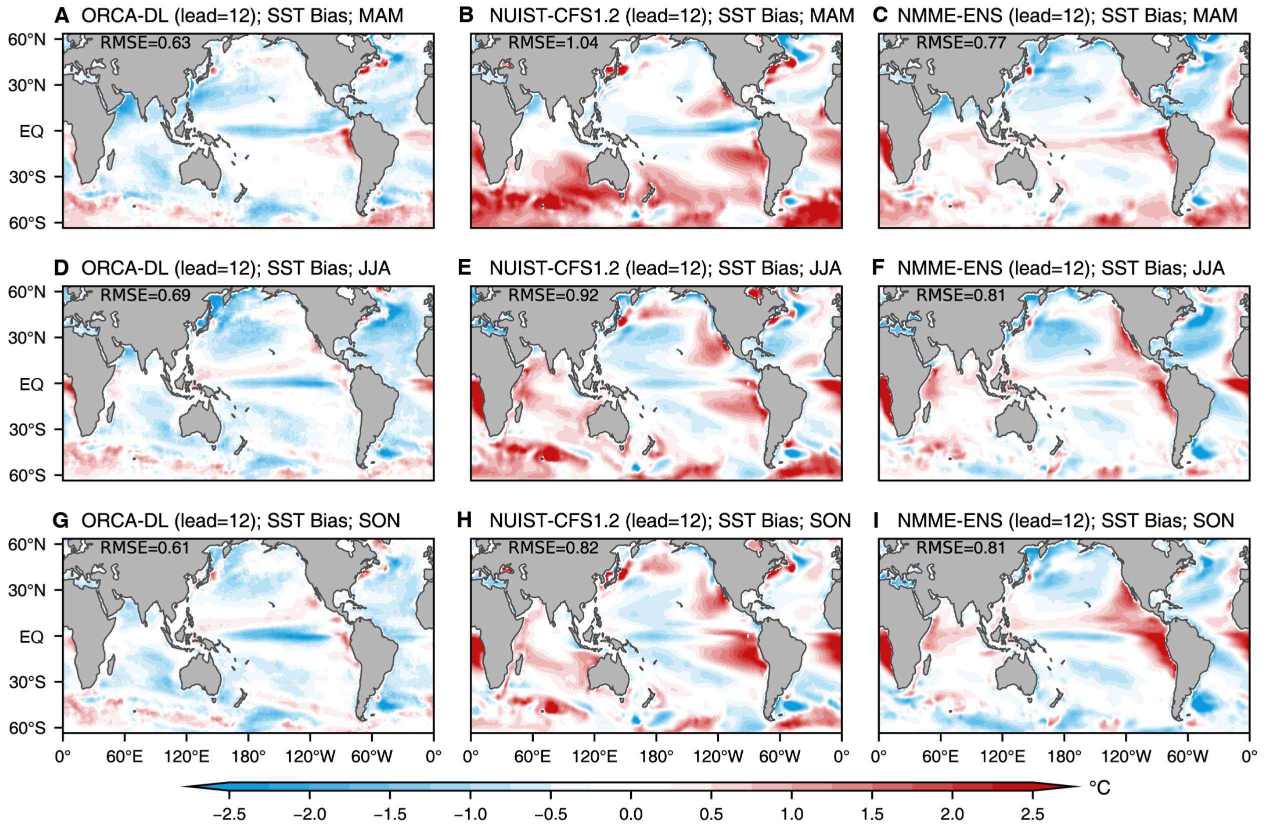

Fig. S2. The bias of SST mean states. (**A** to **C**) The bias of mean states in MAM season for ORCA-DL, NUIST-CFS1.2 and NMME-ENS, respectively. All models use a 12-month lead time forecast. (**D** to **F**) and (**G** to **I**) As in (A) to (C), but for JJA and SON seasons, respectively.

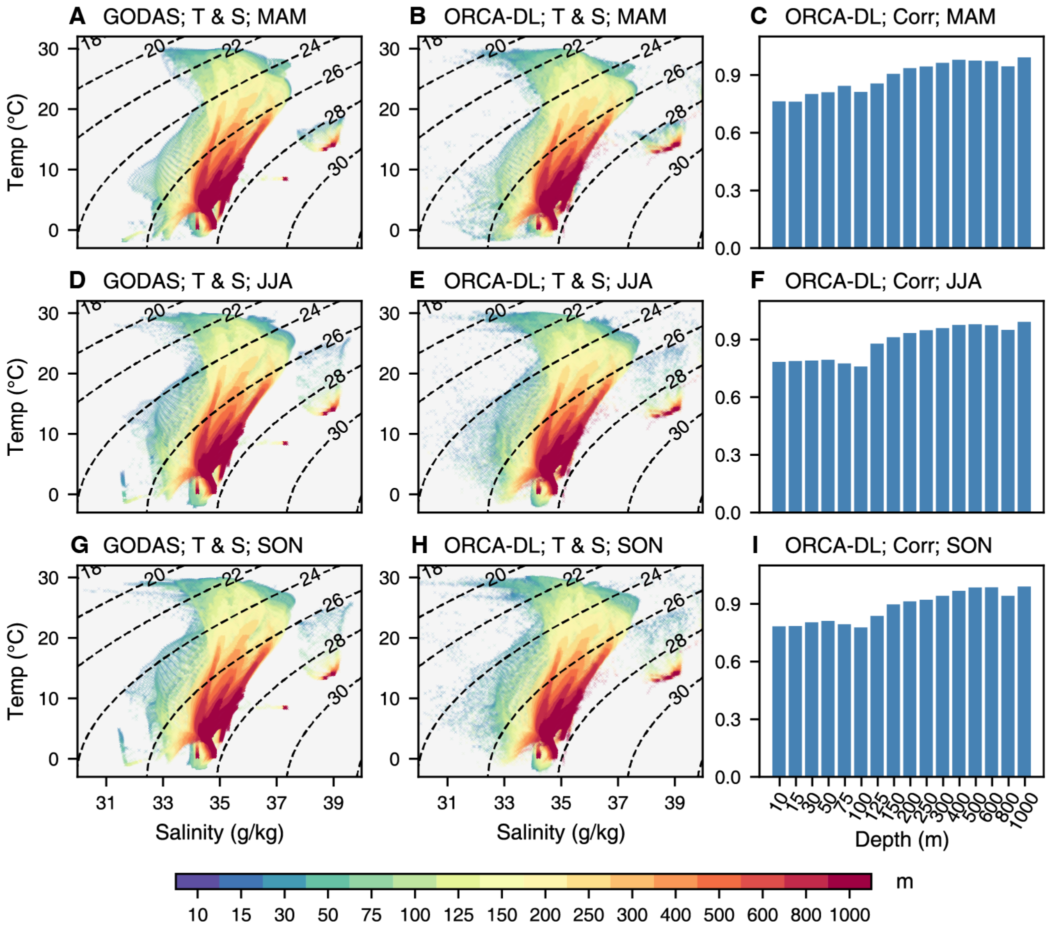

Fig. S3. The scatter plots of the temperature and salinity mean states. (**A** to **B**) The plots in MAM season based on GODAS and ORCA-DL's predictions at 12 months lead, respectively. The dashed contour lines denote potential density with a reference pressure of 0 dbar (σ_0 density, kg/m^3). The color of points represents depth. (**C**) Correlation for temperature-salinity distribution between ORCA-DL (as shown in (A)) and GODAS (as shown in (B)) at each depth. (**D** to **F**) and (**G** to **I**) As in (A) to (C), but for JJA and SON seasons, respectively.

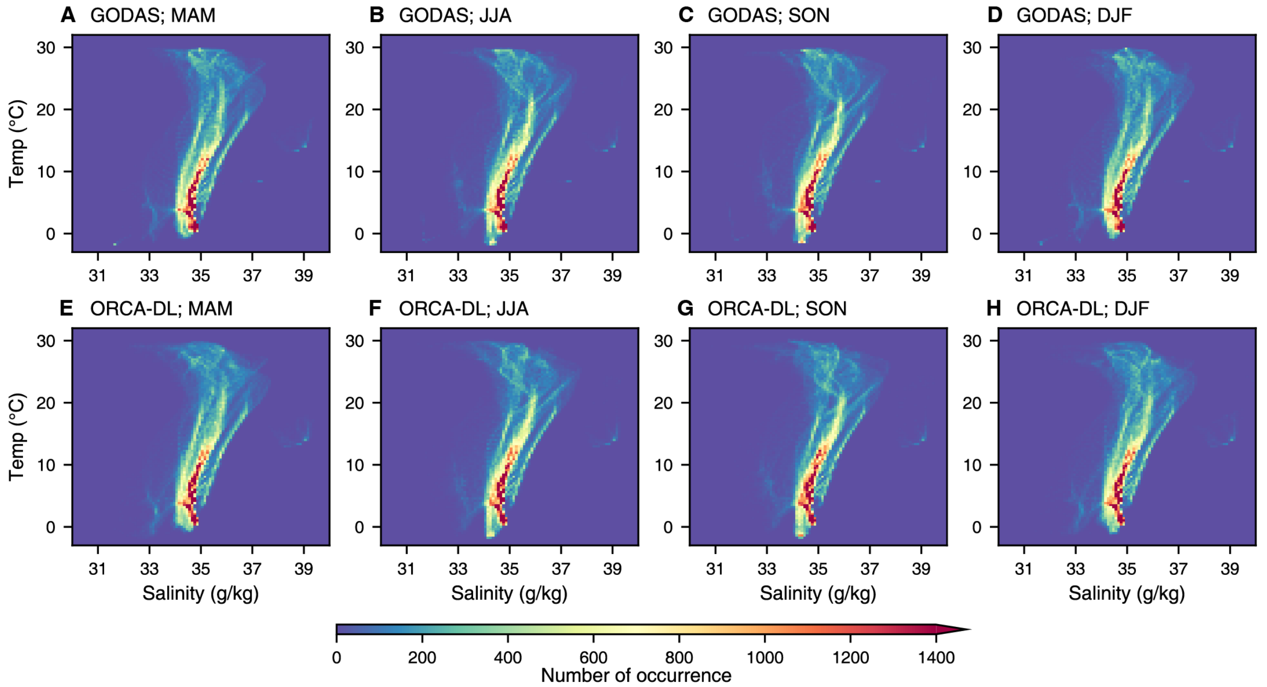

Fig. S4. Density distribution of surface temperature-salinity points. (**A** to **D**) The distribution based on GODAS in different seasons during 1985-2018 respectively. The x and y axes are divided into 100 small intervals respectively, making a total of 100×100 grids, and the number of points falling in each grid is counted. (**E** to **H**) As in (A) to (D), but for ORCA-DL predictions at a lead time of 12 months.

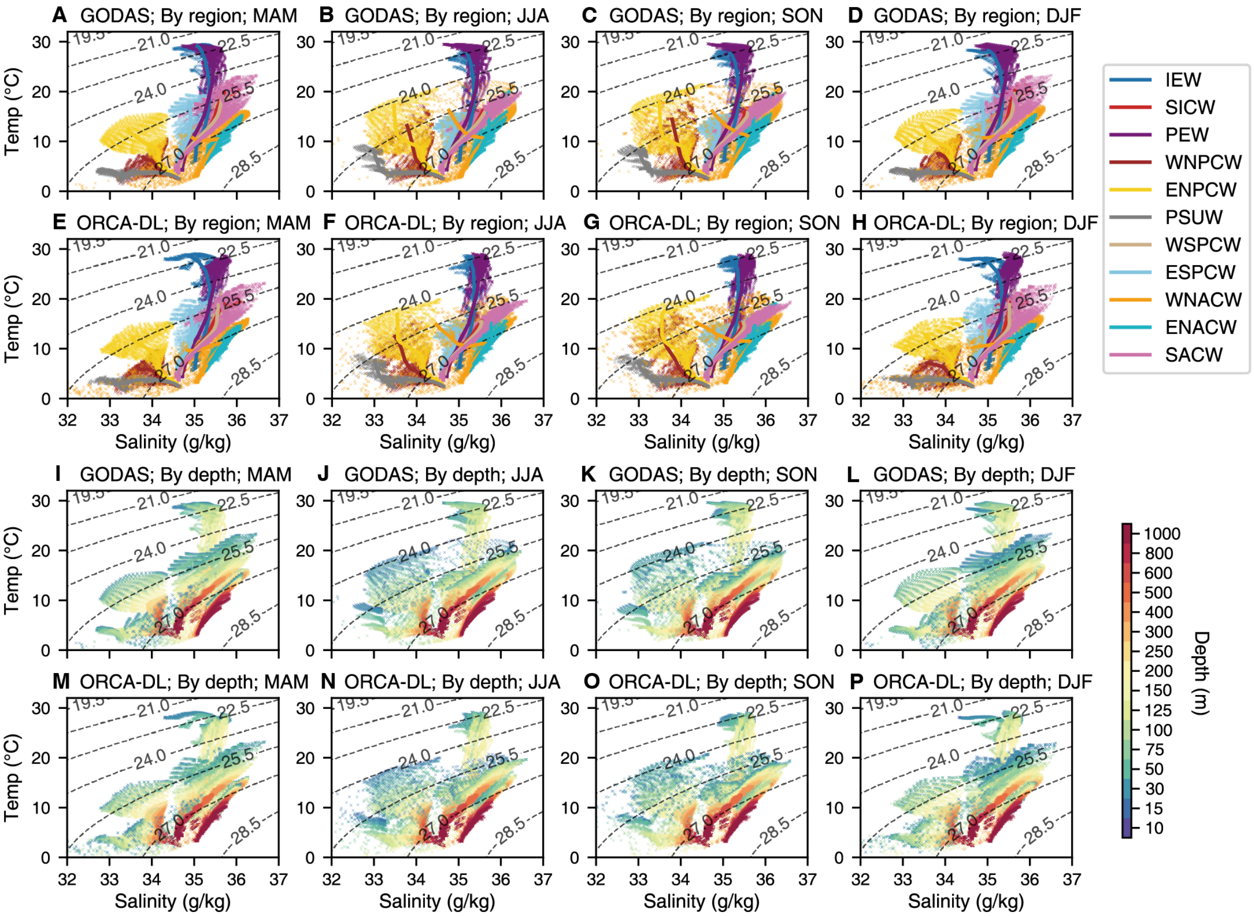

Fig. S5. Temperature-Salinity plot in different water masses. (**A** to **D**) The temperature-salinity plot based on the climatology of GODAS at four seasons. respectively. The testing period is 1985-2018. The points in different regions are displayed in different colors. The water masses include the Indian Ocean Equatorial Water (IEW), South Indian Ocean Central Water (SICW), Pacific Equatorial Water (PEW), Western North Pacific Central Water (WNPCW), Eastern North Pacific Central Water (ENPCW), Pacific Subarctic Upper Water (PSUW), Western South Pacific Central Water (WSPCW), Eastern South Pacific Central Water (ESPCW), Western North Atlantic Central Water (WNACW), Eastern North Atlantic Central Water (ENACW), and South Atlantic Central Water (SACW). The dashed contour lines denote potential density with a reference pressure of 0 dbar (σ_0 density, kg/m^3). (**E** to **H**) As in (A) to (D), but for ORCA-DL at a lead time of 12 months. (**I** to **P**) As in (A) to (H), but colored by depth.

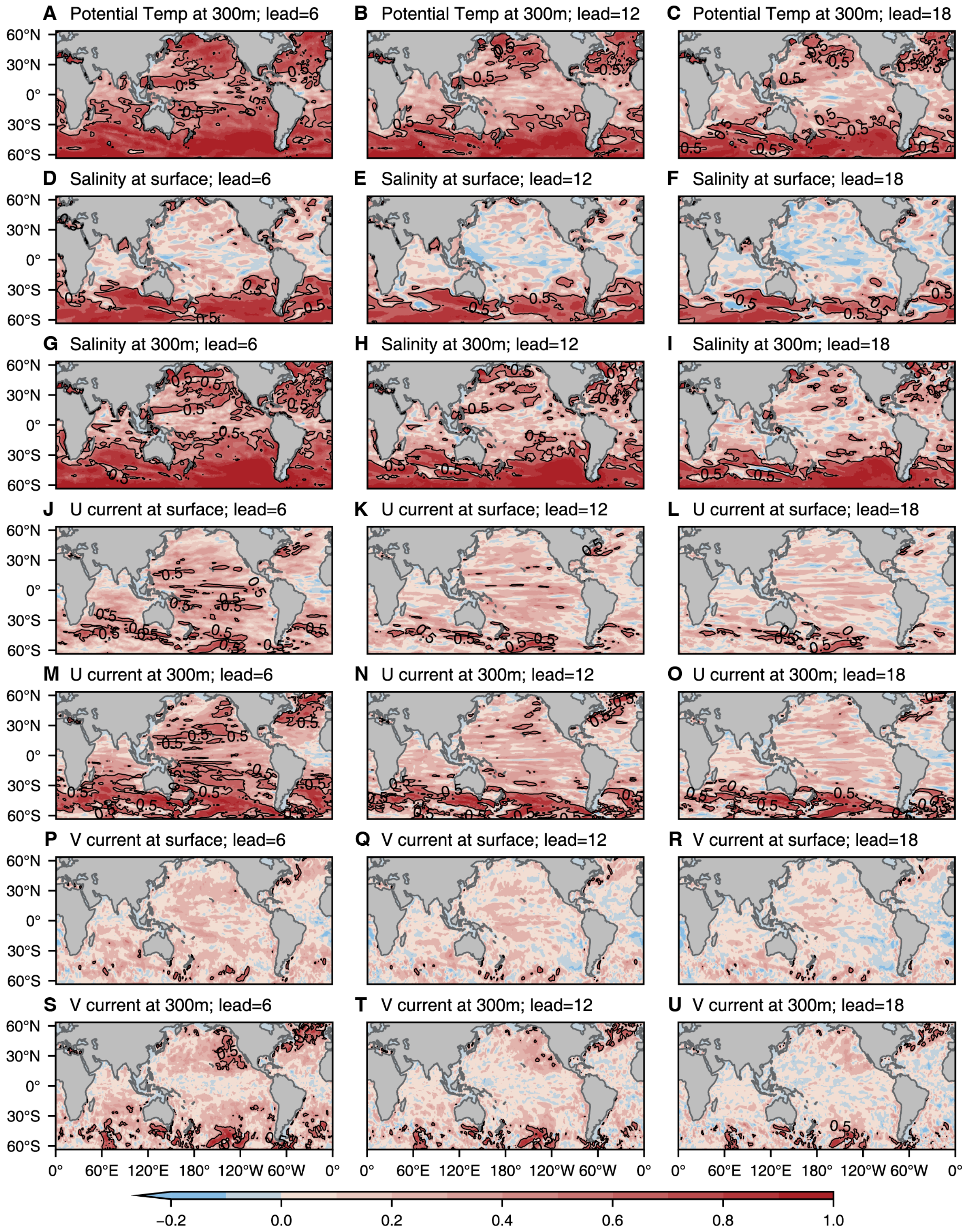

Fig. S6. Distributions of Temporal Correlation Coefficient (TCC) skills for ORCA-DL. (**A** to **C**) TCC skills for prediction of potential temperature anomaly at 300m depth at lead time of 6, 12, 18 months, respectively. (**D** to **F**), (**G** to **I**), (**J** to **L**), (**M** to **O**), (**P** to **R**) and (**S** to **U**) As in (A) to (C), but for salinity at surface, salinity at 300m depth, zonal current at surface, zonal current at 300m depth, meridional current at surface, meridional current at 300m depth.

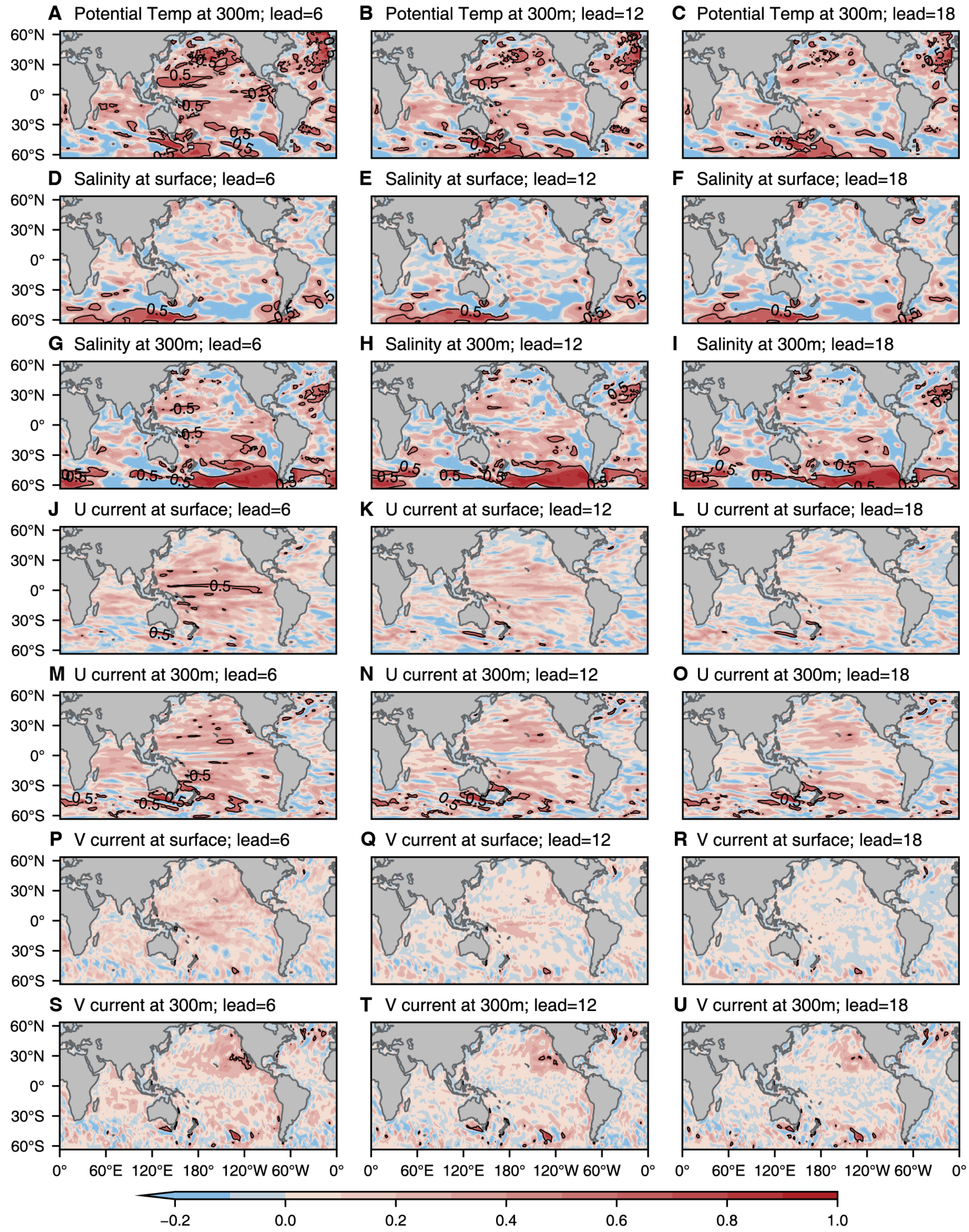

Fig. S7. Distributions of TCC skills for NUIST-CFS1.2. (**A** to **C**) TCC skills for prediction of potential temperature anomaly at 300m depth at lead time of 6, 12, 18 months, respectively. (**D** to **F**), (**G** to **I**), (**J** to **L**), (**M** to **O**), (**P** to **R**) and (**S** to **U**) As in (A) to (C), but for salinity at surface, salinity at 300m depth, zonal current at surface, zonal current at 300m depth, meridional current at surface, meridional current at 300m depth.

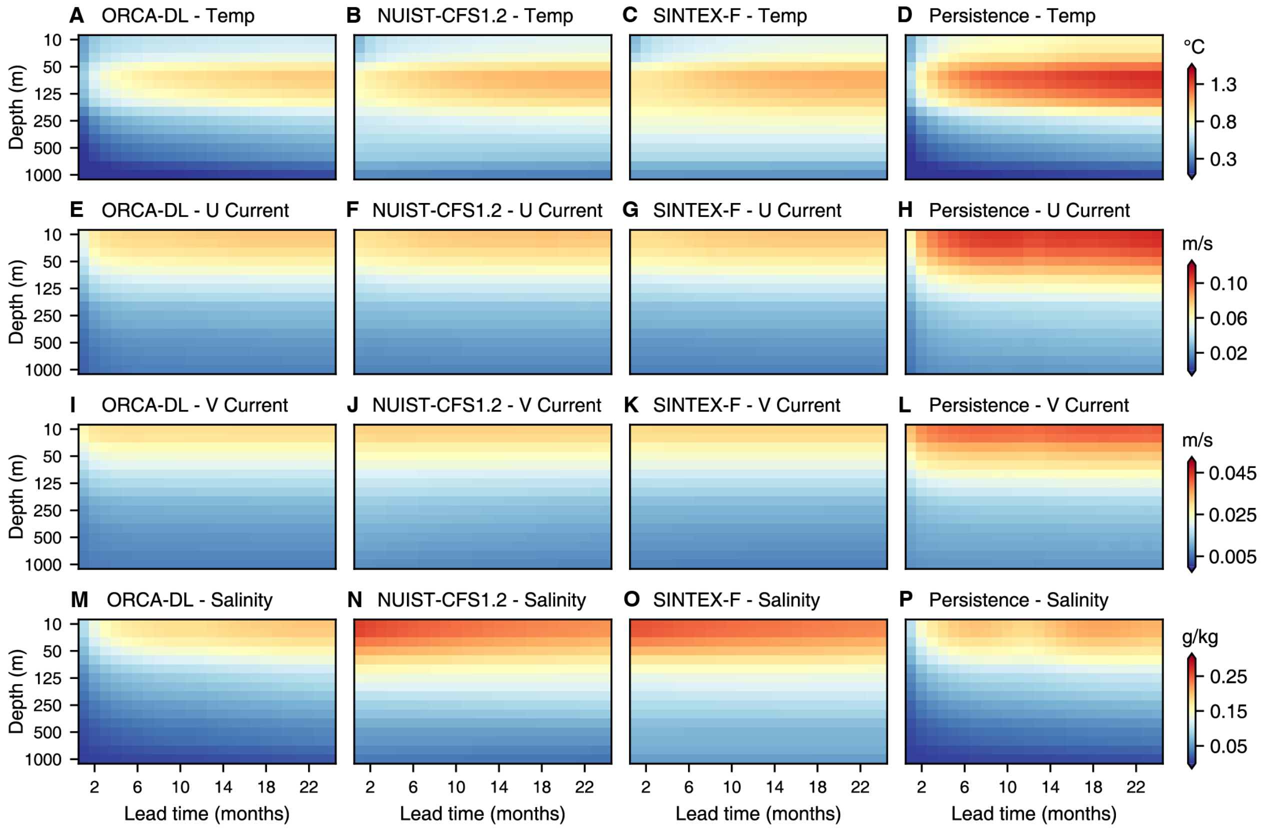

Fig. S8. RMSE skills at each depth. (**A** to **D**) The global averaged RMSE of potential temperature from ORCA-DL, NUIST-CFS1.2, SINTEX-F, and persistence forecast, respectively. The x-axis and y-axis represent the forecast lead time and depth, respectively. (**E** to **P**) As in (A) to (D), but for the RMSE of zonal current, meridional current, and salinity prediction, respectively. All the skills are for anomalies.

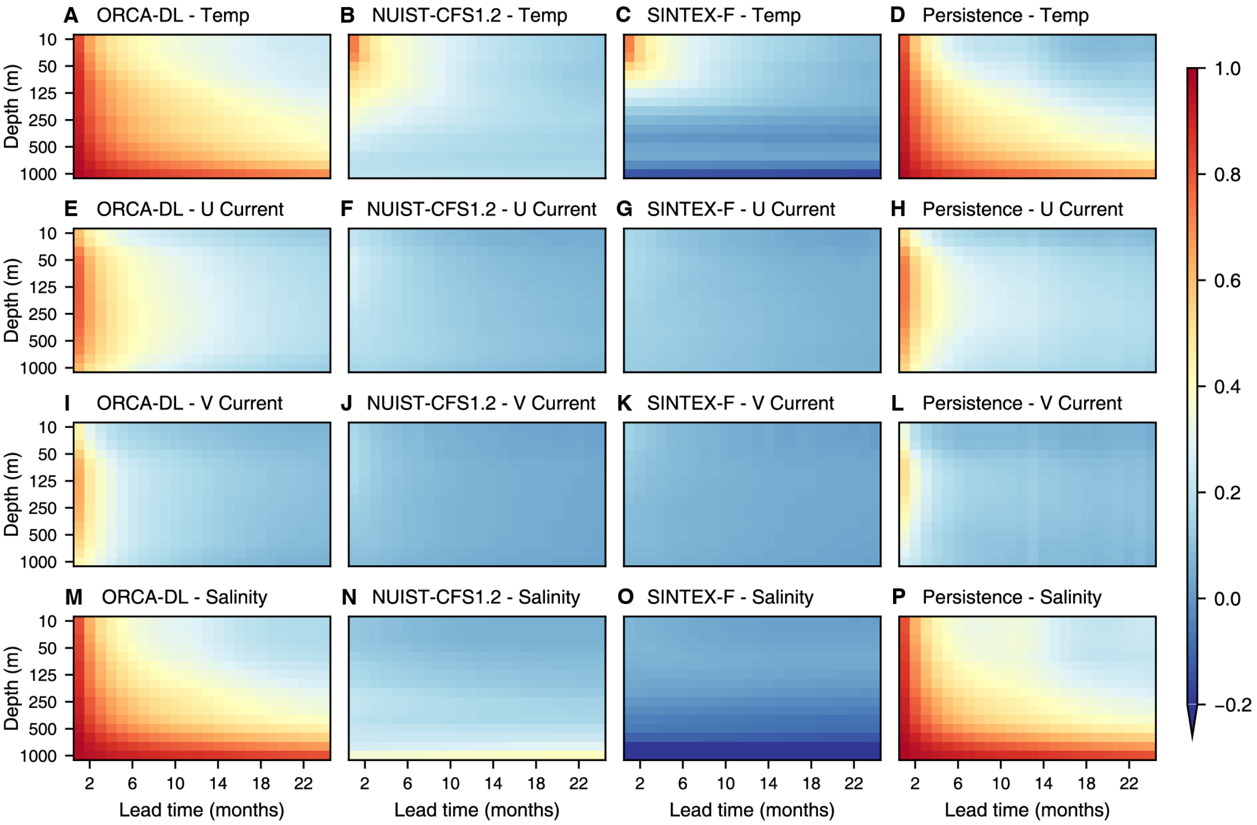

Fig. S9. TCC skills at each depth. (**A** to **D**) The global averaged TCC skills of potential temperature from ORCA-DL, NUIST-CFS1.2, SINTEX-F, and persistence forecast, respectively. The x-axis and y-axis represent the forecast lead time and depth, respectively. (**E** to **P**) As in (A) to (D), but for the TCC skills of zonal current, meridional current, and salinity prediction, respectively.

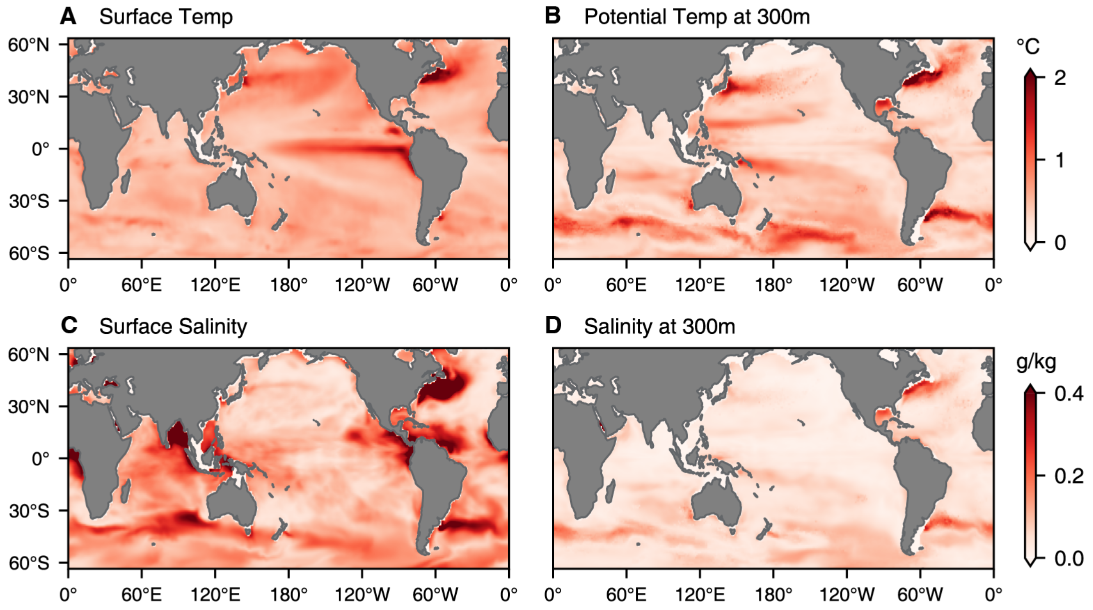

Fig. S10. Distributions of the standard deviation (STD) of anomalies based on GODAS during 1985-2018. (**A** to **B**) The std of temperature anomalies at the surface and 300m, respectively. (**C** to **D**) As in (A) to (B), but for that of salinity anomalies.

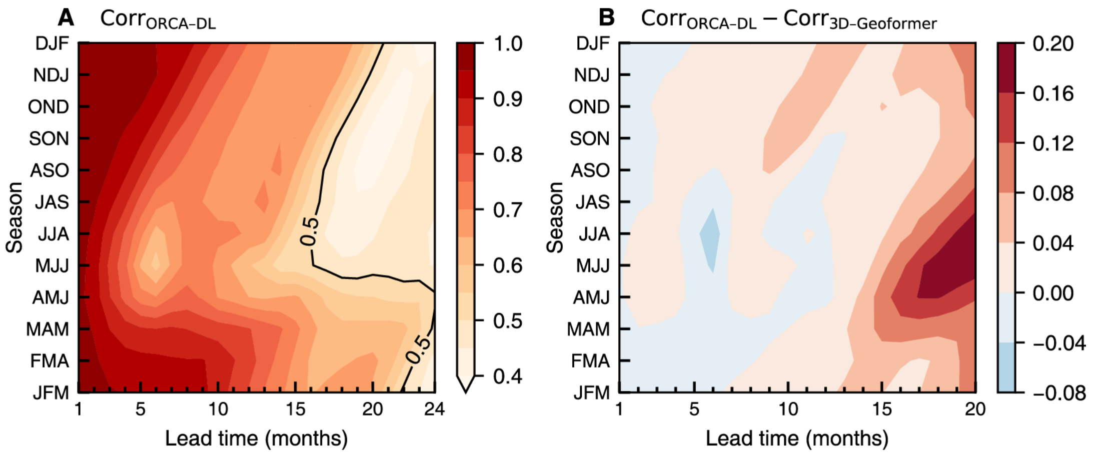

Fig. S11. Niño3.4 index forecast skills for different target season (1985-2018). (**A**) The performance of ORCA-DL for different target seasons. (**B**) The performance differences between ORCA-DL and 3D-Geoformer (*39*).

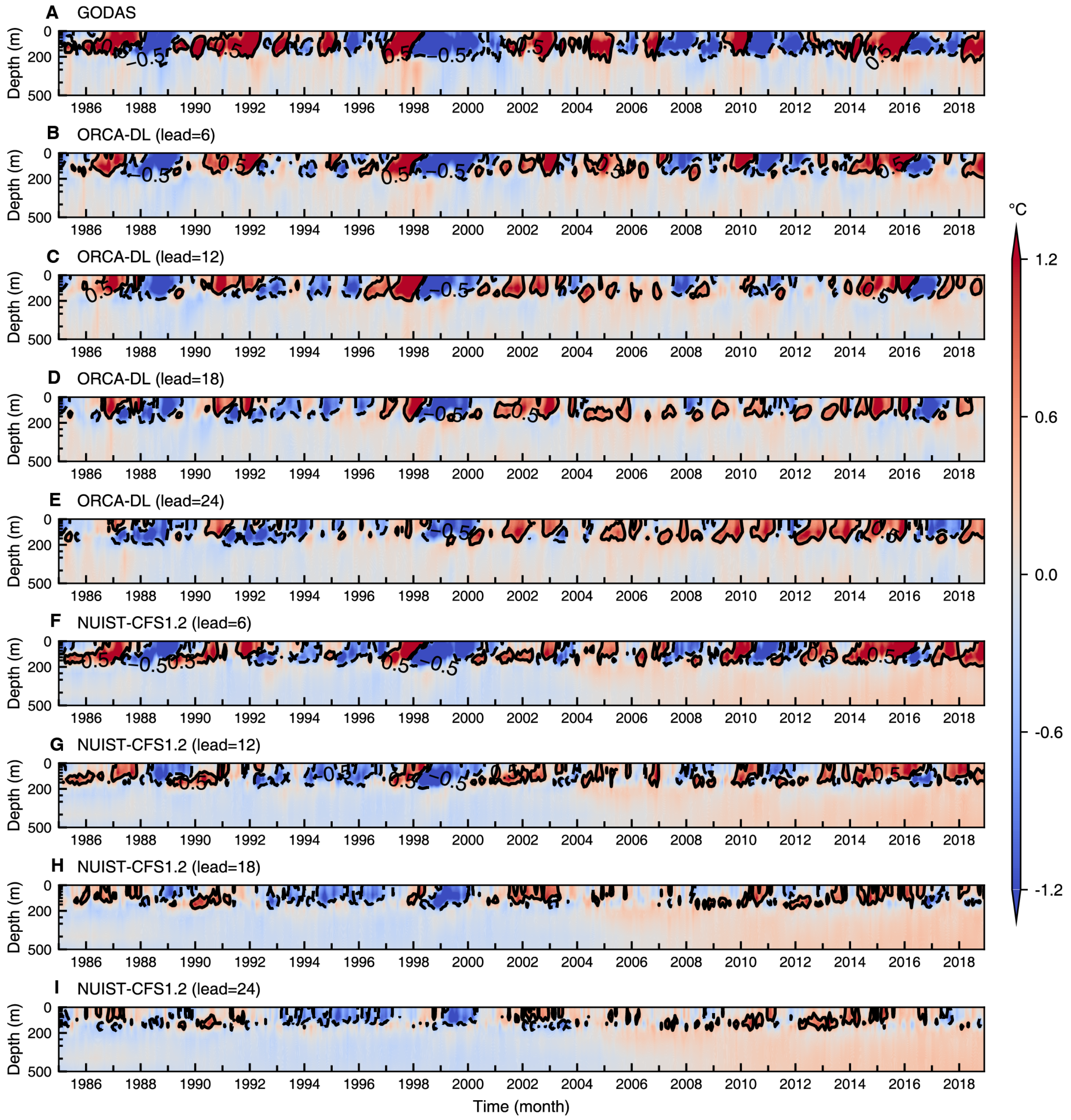

Fig. S12. The depth-temporal evolution of the mean temperature anomalies in Niño3.4 region. (**A** to **I**) The averaged anomalies based on GODAS and predictions of ORCA-DL and NUIST-CFS1.2 with lead times of 6, 12, 18, and 24 months, respectively. The x-axis and y-axis denote the time and depth respectively.

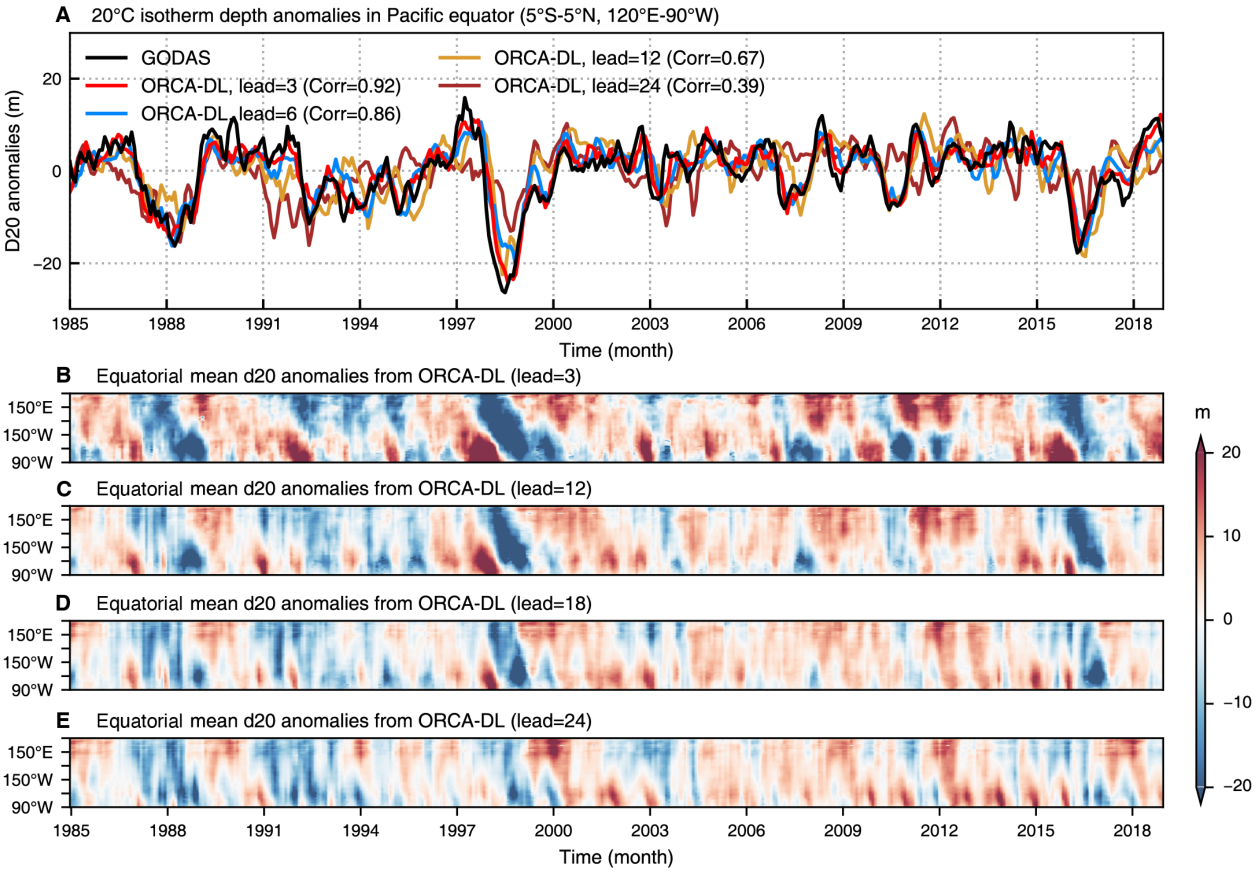

Fig. S13. Prediction of 20°C isotherm depth. (**A**) The 20°C isotherm depth (d20) anomalies averaged over the equatorial Pacific basin (5°S-5°N, 120°E-90°W) based on GODAS and ORCA-DL prediction. (**B** to **E**) As in Fig. 3 (C to D) but for ORCA-DL prediction with lead times of 3, 12, 18, and 24 months, respectively.

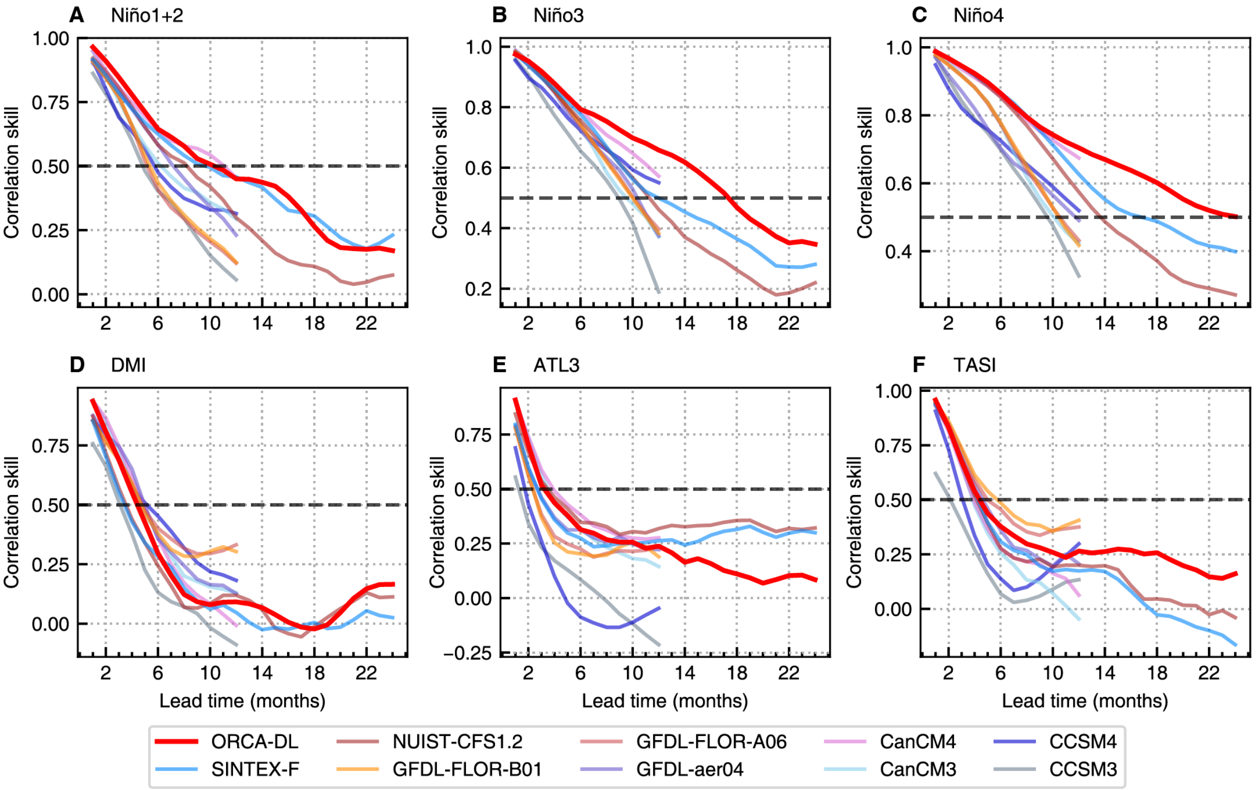

Fig. S14. Forecast skills of major climate indices. (**A** to **F**) The forecast skills for six climate indices as a function of the lead time, including Niño1+2 (10°S-0°, 90°W-80°W), Niño3 (5°S-5°N, 150°W-90°W), Niño4 (5°S-5°N, 160°E-150°W) SST anomaly, the Indian Ocean Dipole index (DMI) (*73*), Atlantic Niño index (ATL3) (*74*), and Tropical Atlantic SST index (TASI) (*75*).

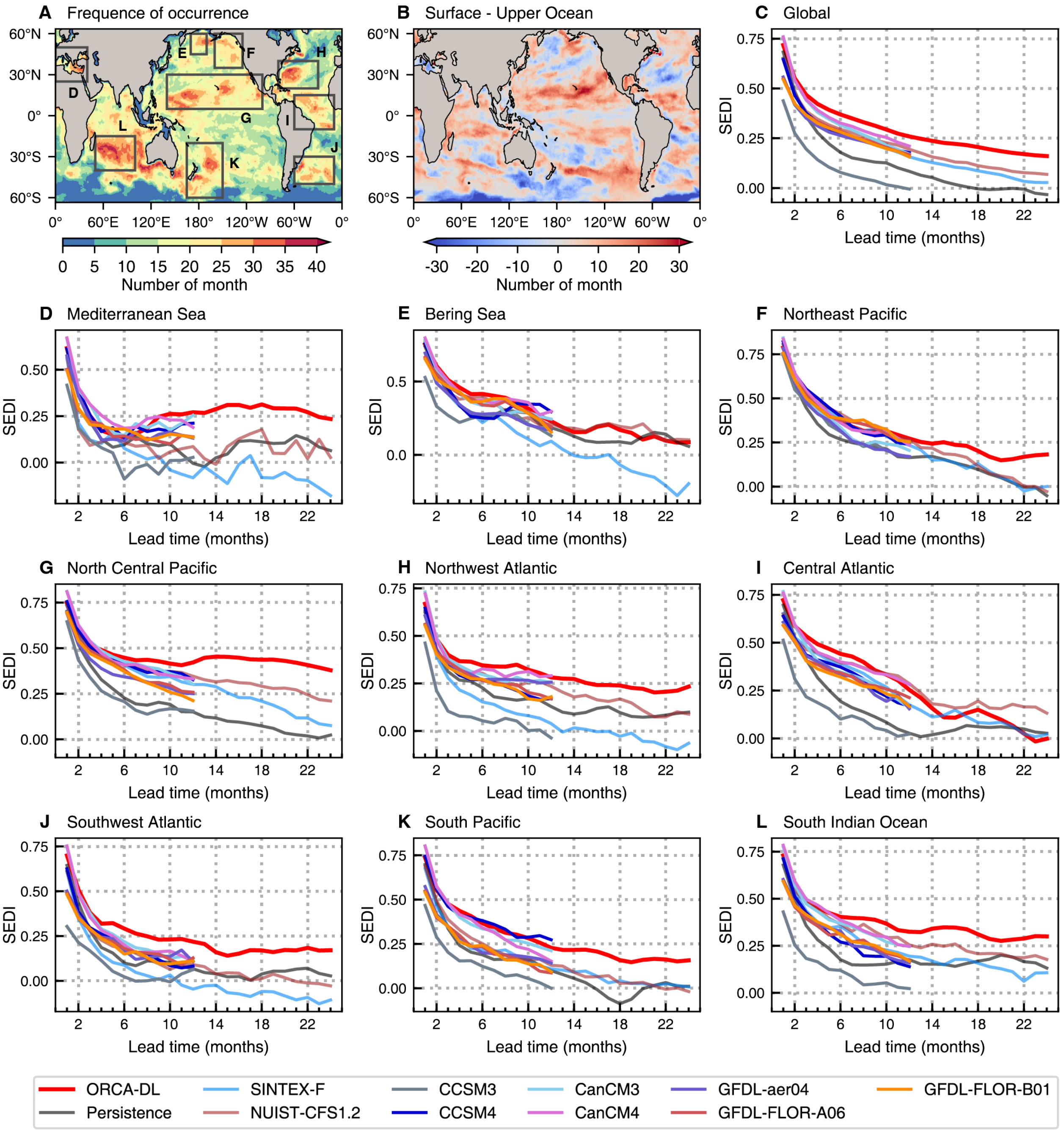

Fig. S15. Forecast skills of surface MHWs. (**A**) The distribution of the frequency of the occurrence of surface MHWs events based on GODAS. (**B**) Differences in the distribution of surface and upper ocean MHWs. (**C** to **L**) The averaged SEDI as a function of the lead time for global and nine regions (Table S2) with a relatively high frequency of heatwave occurrences. Higher SEDI means better skill.

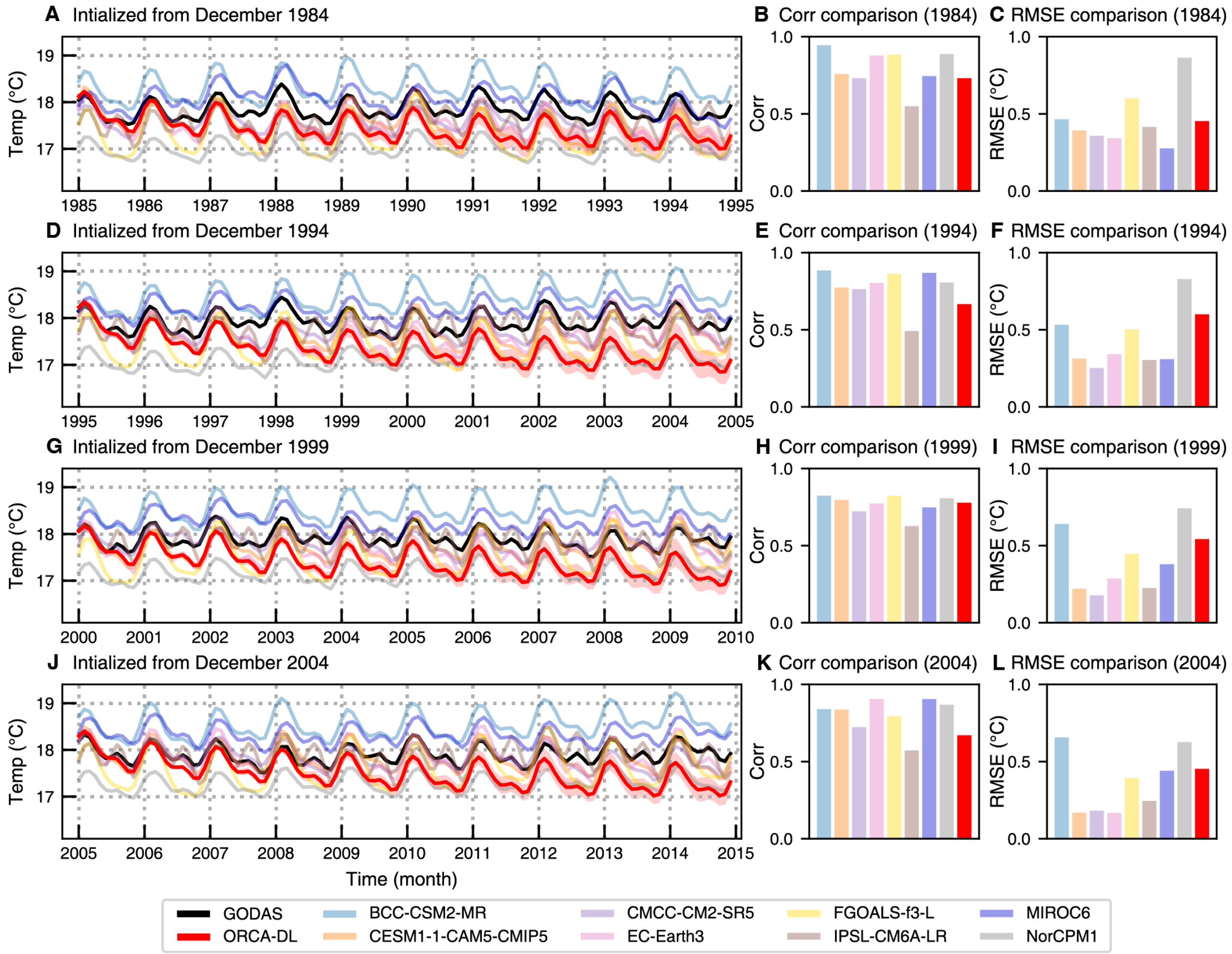

Fig. S16. The global mean SST based on GODAS, and the simulations of ORCA-DL, and eight CMIP6 DCPP models. (**A** to **C**) As in Fig. 5 (A to C) but for that initialized from December 1984. (**D** to **F**), (**G** to **I**) and (**J** to **L**) As in (A) to (C), but for that initialized from December 1994, December 1999 and December 2004, respectively.

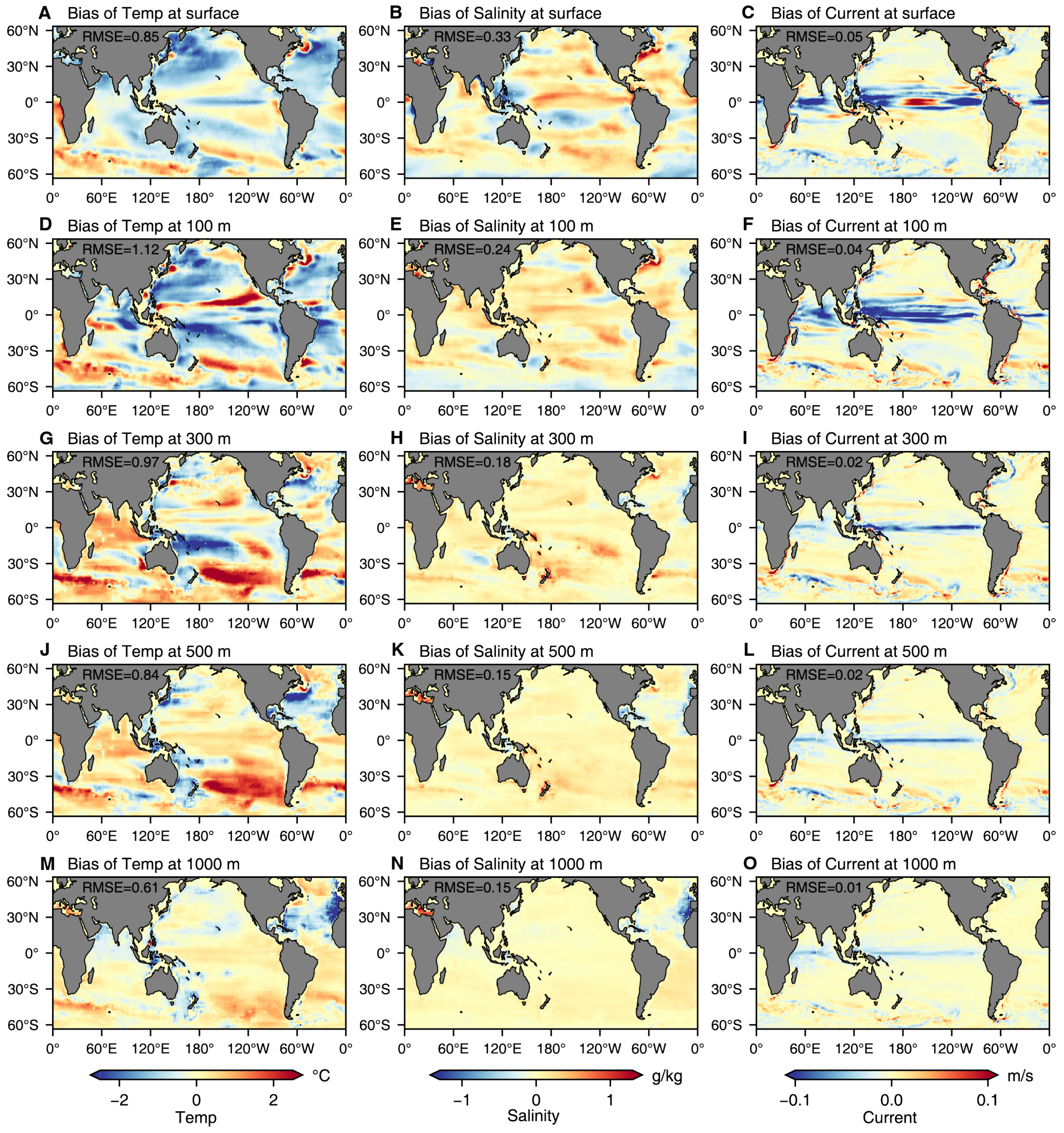

Fig. S17. The mean state bias of the decadal prediction of ORCA-DL. (**A** to **C**) The bias of temperature, salinity, and current at the surface, respectively. The mean state is averaged over the 10-year rollout forecast initialized from December 1989 (1990-1999). (**D** to **F**), (**G** to **I**), (**J** to **L**), and (**M** to **O**) As in (A) to (C), but for the prediction bias at 100m, 300m, 500m, and 1000m depth, respectively.

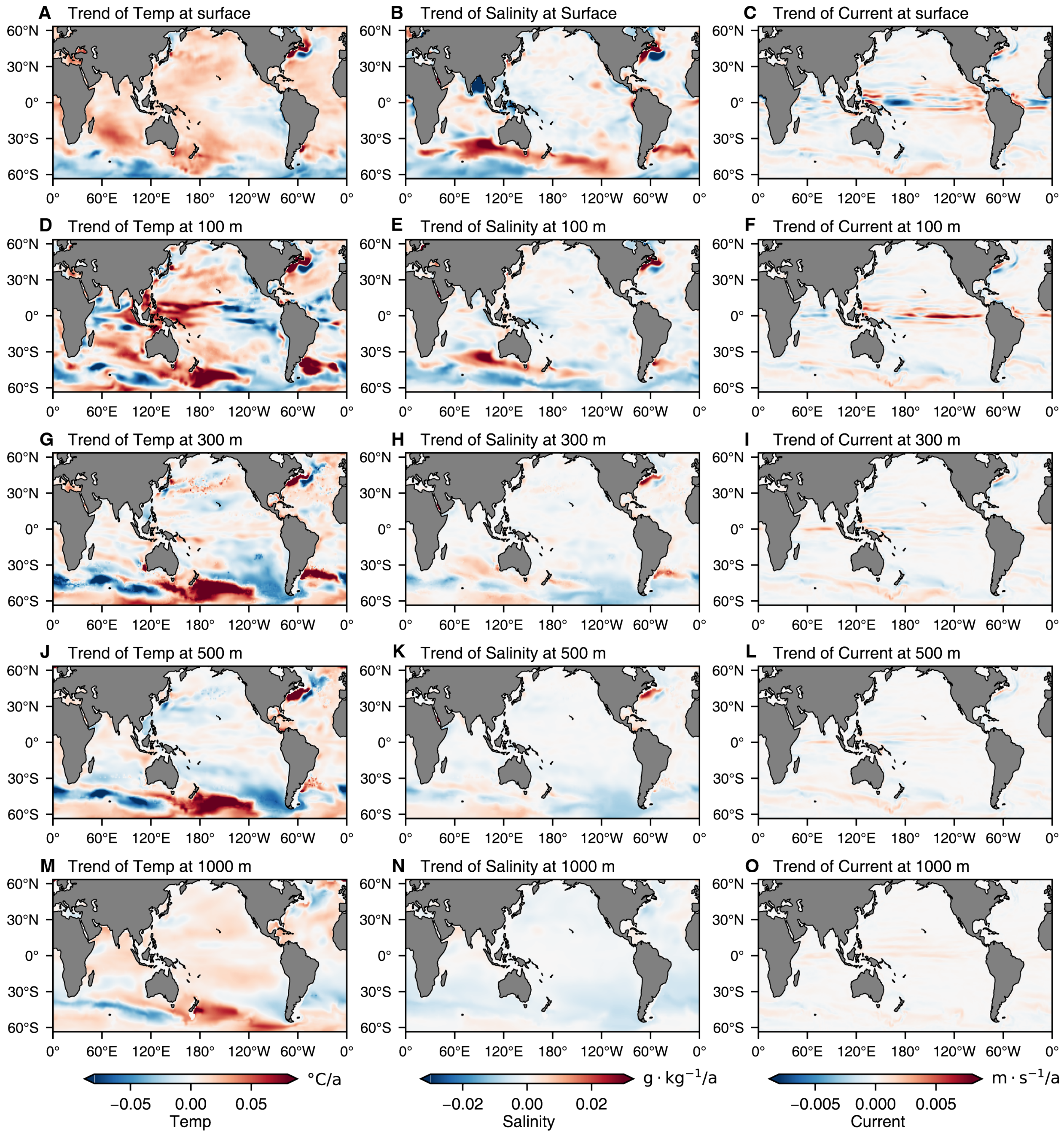

Fig. S18. Trend distribution based on GODAS during 1990-2019. (**A** to **C**) The multi-decadal trend of temperature, salinity, and current at surface, respectively. The trend is the slope coefficient of the least squares method and is calculated based on the annual mean data of GODAS (1990-2019). (**D** to **F**), (**G** to **I**), (**J** to **L**), and (**M** to **O**) As in (A) to (C), but for the trends at 100m, 300m, 500m, and 1000m depth, respectively.

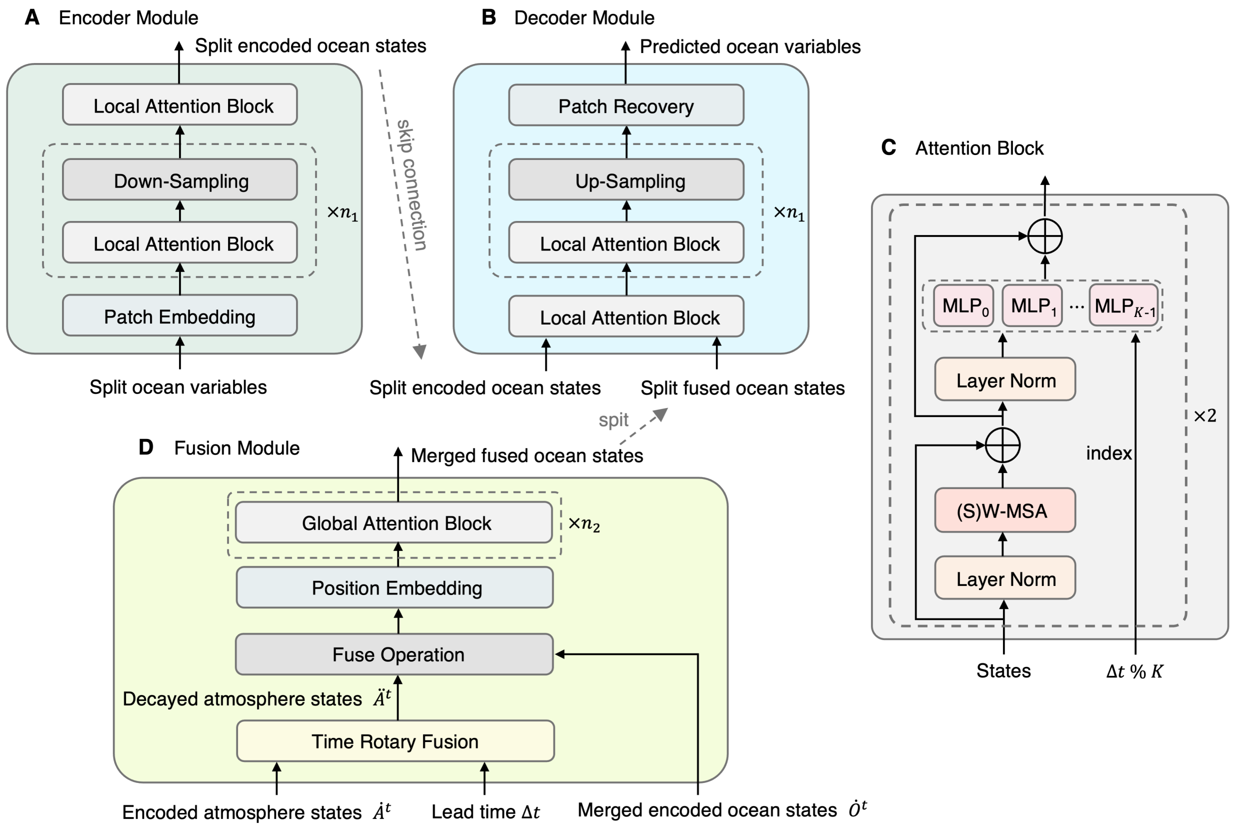

Fig. S19. Detailed structures of ORCA-DL. (**A**) The encoder module consists of a Patch Embedding layer and several attention blocks to encode the input variables. (**B**) The decoder module has an inverse process like encoder. The output of the encoder is also fed into the decoder via skip connection (not shown in Fig. 6). (**C**) The attention block contains two successive sub-blocks. The first sub-block employs window-based multi-head self-attention (W-MSA) to capture the local information, while the subsequent sub-block uses shifted W-MSA (SW-MSA) for interactions across the windows. The lead time Δt determines the prediction interval by selecting the sub-network (MLP) in attention block. The interval is K if the remainder ($\Delta t \,\%\, K$) is 0, otherwise the remainder is the interval. (**D**) The fusion module first uses a Time Rotary Fusion layer to decay the impact of encoded atmosphere states and then fuse the output with encoded ocean states. After that, a Position Embedding layer is used to add a global location information. Finally, several global attention blocks are used for extract high-dimensional signals.

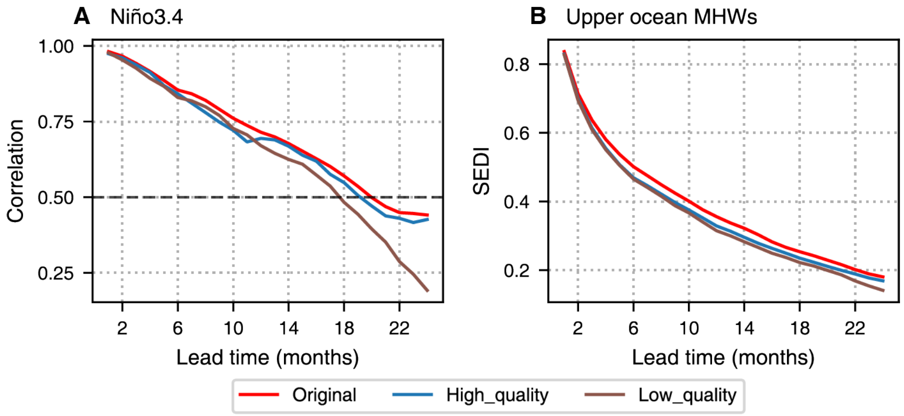

Fig. S20. Contribution of the CMIP6 models to ORCA-DL's skills. (**A** to **B**) Skills comparison for Niño3.4 index prediction and upper ocean marine heat waves prediction, respectively. 'Original' (red lines) denotes the original ORCA-DL, 'High_quality' (blue line) denotes ORCA-DL trained on 10 relatively high-quality CMIP6 models, and 'Low_quality' (brown line) denotes ORCA-DL trained on 10 relatively low-quality CMIP6 models.

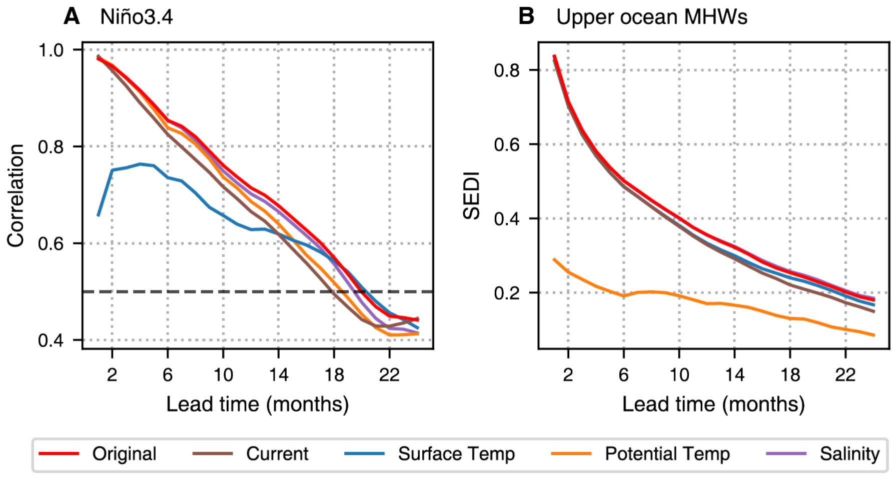

Fig. S21. Compare the performance of replacing different variables with climate states. (**A** to **B**) Skills comparison for Niño3.4 index prediction and upper ocean marine heat waves prediction, respectively. 'Original' (red lines) denotes the original ORCA-DL, and other colored lines represent the predictions made by ORCA-DL after replacing different variables in the initial field with corresponding climate mean states.

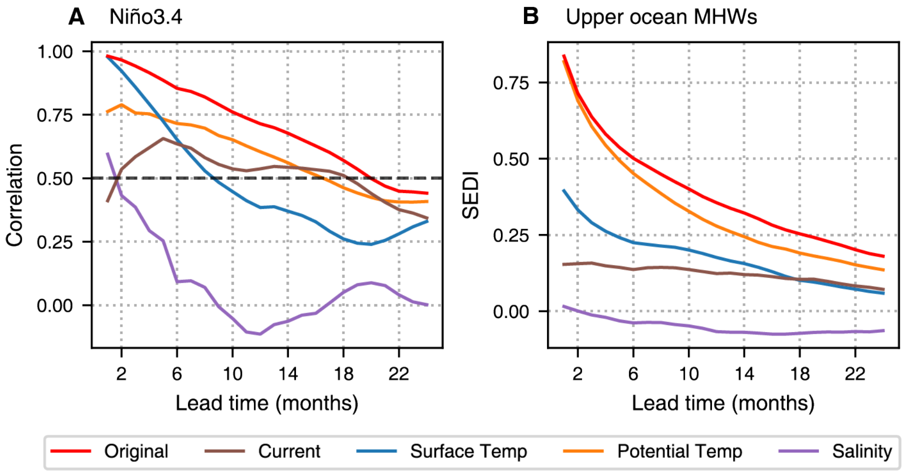

Fig. S22. Compare the performance of retaining one variable and replacing the others with climatic states. (**A** to **B**) Skills comparison for Niño3.4 index prediction and upper ocean marine heat waves prediction, respectively. 'Original' (red lines) denotes the original ORCA-DL, and other colored lines represent predictions made by ORCA-DL after keeping only one variable in the initial field and replacing the other variables with their climate mean states, respectively.

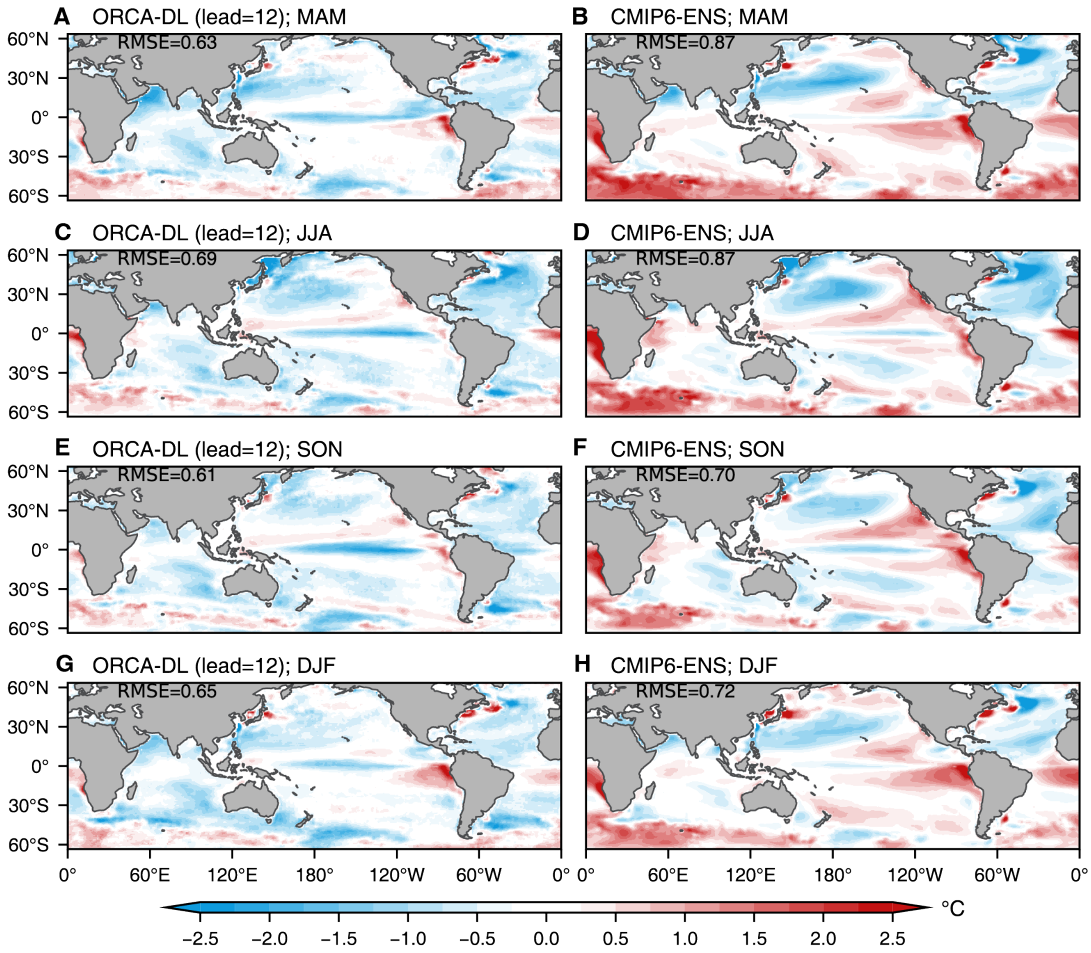

Fig. S23. Comparison of mean states biases of SST between ORCA-DL and CMIP6-ENS. The first column is the bias of ORCA-DL prediction at 12-month lead in four different seasons, while the second column shows the bias of CMIP6-ENS. CMIP6-ENS denotes the ensemble mean of 20 CMIP6 models used for training ORCA-DL. The global ocean mean RMSE is displayed in each panel.

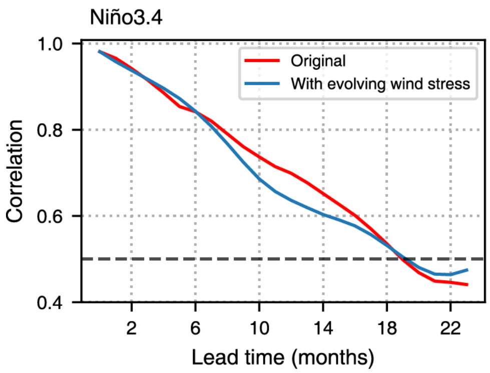

Fig. S24. Performance of inputting instantaneous wind stress on ENSO forecast.

Table S1. Variables and vertical levels modeled by ORCA-DL. The input oceanic variables consist of two surface variables and four multi-layer variables with 16 layers. The input atmospheric variables only include two surface variables as initial conditions and are not predicted by ORCA-DL.

	Surface variables	Multi-layer variables	Vertical levels
ocean	surface temperature<br>surface height above the geoid	potential temperature<br>salinity<br>zonal velocity<br>meridional velocity	10, 15, 30, 50<br>75, 100, 125, 150,<br>200, 250, 300, 400,<br>500, 600, 800, 1000m
atmosphere	zonal wind stress<br>meridional wind stress	/	

Table S2. The range of each region considered for MHWs forecast. The North Central Pacific and Central Atlantic regions are only used for surface MHWs.

Region	Range
Northeast Pacific	(35°N-60°N, 160°W-125°W)
North Central Pacific	(5°N-30°N, 140°E-100°W)
South Pacific	(60°S-20°S, 165°E-150°W)
Northwest Atlantic	(20°N-40°N, 80°W-30°W)
Central Atlantic	(10°S-15°N, 60°W-10°W)
Southwest Atlantic	(50°S-30°S, 60°W-10°W)
South Indian Ocean	(40°S-15°S, 50°E-100°E)
Mediterranean Sea	(25°N-50°N, 0°E-40°E)
Bering Sea	(45°N-60°N, 170°E-170°W)

Table S3. CMIP6 models used for training.

Source ID	Institution
BCC-CSM2-MR	Beijing Climate Center
CAS-ESM2-0	Chinese Academy of Sciences
CIESM	Department of Earth System Science, Tsinghua University
CMCC-CM2-HR4	Fondazione Centro Euro-Mediterraneo sui Cambiamenti Climatici
CMCC-CM2-SR5	Fondazione Centro Euro-Mediterraneo sui Cambiamenti Climatici
CMCC-ESM2	Fondazione Centro Euro-Mediterraneo sui Cambiamenti Climatici
E3SM-1-0	E3SM-Project
E3SM-1-1	E3SM-Project
E3SM-2-0	E3SM-Project
EC-Earth3	EC-Earth Consortium
EC-Earth3-Veg	EC-Earth Consortium
FGOALS-f3-L	Chinese Academy of Sciences
FIO-ESM-2-0	First Institute of Oceanography, Ministry of Natural Resources / Qingdao National Laboratory for Marine Science and Technology
HadGEM3-GC31-MM	Met Office Hadley Centre
INM-CM4-8	Institute for Numerical Mathematics, Russian Academy of Science
INM-CM5-0	Institute for Numerical Mathematics, Russian Academy of Science
MPI-ESM1-2-HR	Max Planck Institute for Meteorology / Deutscher Wetterdienst / Deutsches Klimarechenzentrum
MRI-ESM2-0	Meteorological Research Institute
SAM0-UNICON	Seoul National University
TaiESM1	Research Center for Environmental Changes, Academia Sinica

Table S4. Datasets used for training, validation, and testing.

	Data	Period
Training	CMIP6	1850-2014
Validation	SODA2	1871-1979
	ORAS5	1958-1979
Testing	GODAS	1980-2019

Table S5. Rank of CMIP6 models. The model ranks based on the average of Rank in (*70*) (based on SST bias), Rank in (*71*) (based on ENSO phase-locking bias), and Rank in (*72*) (based on MHW projection).

CMIP6 Model	Rank in [66]	Rank in [67]	Rank in [68]	Overall Rank
BCC-CSM2-MR	8	14	8	14
CAS-ESM2-0	12	10	/	16
CIESM	5	/	/	3
CMCC-CM2-HR4	6	/	2	2
CMCC-CM2-SR5	10	4	1	4
CMCC-ESM2	7	/	/	11
E3SM-1-0	/	7	/	10
E3SM-1-1	/	12	/	19
E3SM-2-0	/	/	/	20
EC-Earth3	14	8	/	17
EC-Earth3-Veg	11	6	6	12
FGOALS-f3-L	3	9	7	6
FIO-ESM-2-0	1	2	3	1
HadGEM3-GC31-MM	/	/	10	15
INM-CM4-8	/	11	/	18
INM-CM5-0	/	5	/	5
MPI-ESM1-2-HR	2	13	4	7
MRI-ESM2-0	4	15	5	13
SAM0-UNICON	9	3	9	8
TaiESM1	13	1	/	9

REFERENCES AND NOTES

1. A. Troccoli, Seasonal climate forecasting. *Meteorol. Appl.* **17**, 251–268 (2010).

2. G. A. Meehl, J. H. Richter, H. Teng, A. Capotondi, K. Cobb, F. Doblas-Reyes, M. G. Donat, M. H. England, J. C. Fyfe, W. Han, H. Kim, B. P. Kirtman, Y. Kushnir, N. S. Lovenduski, M. E. Mann, W. J. Merryfield, V. Nieves, K. Pegion, N. Rosenbloom, S. C. Sanchez, A. A. Scaife, D. Smith, A. C. Subramanian, L. Sun, D. Thompson, C. C. Ummenhofer, S.-P. Xie, Initialized earth system prediction from subseasonal to decadal timescales. *Nat. Rev. Earth Environ.* **2**, 340–357 (2021).

3. D. A. Randall, *General Circulation Model Development: Past, Present, and Future* (Elsevier, 2000).

4. F. Boberg, J. Christensen, Overestimation of Mediterranean summer temperature projections due to model deficiencies. *Nat. Clim. Change* **2**, 433–436 (2012).

5. J. Shukla, J. Anderson, D. Baumhefner, C. Brankovic, Y. Chang, E. Kalnay, L. Marx, T. Palmer, D. Paolino, J. Ploshay, S. Schubert, D. Straus, M. Suarez, J. Tribbia, Dynamical seasonal prediction. *Bull. Am. Meteorol. Soc.* **81**, 2593–2606 (2000).

6. M. Kanamitsu, A. Kumar, H.-M. H. Juang, J.-K. Schemm, W. Wang, F. Yang, S.-Y. Hong, P. Peng, W. Chen, S. Moorthi, M. Ji, NCEP dynamical seasonal forecast system 2000. *Bull. Am. Meteorol. Soc.* **83**, 1019–1038 (2002).

7. C. Wang, Three-ocean interactions and climate variability: A review and perspective. *Clim. Dyn.* **53**, 5119–5136 (2019).

8. K. Hasselmann, An ocean model for climate variability studies. *Prog. Oceanogr.* **11**, 69–92 (1982).

9. B. P. Kirtman, T. Stockdale, R. Burgman, “The ocean’s role in modeling and predicting seasonal-to-interannual climate variations” in *Ocean Circulation and Climate*, vol. 103 of *International Geophysics* (Elsevier, 2013), pp. 625–643.

10. S. M. Griffies, C. Böning, F. O. Bryan, E. P. Chassignet, R. Gerdes, H. Hasumi, A. Hirst, A.-M. Treguier, D. Webb, Developments in ocean climate modelling. *Ocean Model.* **2**, 123–192 (2000).

11. T. N. Palmer, G. J. Shutts, R. Hagedorn, F. J. Doblas-Reyes, T. Jung, M. Leutbecher, Representing model uncertainty in weather and climate prediction. *Annu. Rev. Earth Planet. Sci.* **33**, 163–193 (2005).

12. P. N. Edwards, History of climate modeling. *Wiley Interdiscip. Rev. Clim. Change* **2**, 128–139 (2011).

13. T. Schneider, S. Behera, G. Boccaletti, C. Deser, K. Emanuel, R. Ferrari, L. R. Leung, N. Lin, T. Müller, A. Navarra, O. Ndiaye, A. Stuart, J. Tribbia, T. Yamagata, Harnessing AI and computing to advance climate modelling and prediction. *Nat. Clim. Change* **13**, 887–889 (2023).

14. T. Barnett, R. Preisendorfer, Origins and levels of monthly and seasonal forecast skill for United States surface air temperatures determined by canonical correlation analysis. *Mon. Weather Rev.* **115**, 1825–1850 (1987).

15. M. Newman, Interannual to decadal predictability of tropical and North Pacific sea surface temperatures. *J. Clim.* **20**, 2333–2356 (2007).

16. J. Schlore, M. Newman, J. Thuemmel, A. Capotondi, B. Goswami, A hybrid deep-learning model for El Niño Southern Oscillation in the low-data regime. arXiv:2412.03743 [cs.LG] (2025).

17. A. Hannachi, I. T. Jolliffe, D. B. Stephenson, Empirical orthogonal functions and related techniques in atmospheric science: A review. *Int. J. Climatol.* **27**, 1119–1152 (2007).

18. T. Frederikse, T. Lee, O. Wang, B. Kirtman, E. Becker, B. Hamlington, D. Limonadi, D. Waliser, A hybrid dynamical approach for seasonal prediction of sea-level anomalies: A pilot study for Charleston, South Carolina. *J. Geophys. Res. Oceans* **127**, e2021JC018137 (2022).

19. C. Finan, H. Wang, J. Schemm, “CFSv2-based hybrid dynamical-statistical model for week 3 to 4 forecast of Atlantic/Pacific tropical storm activity,” in *Climate Prediction S&T Digest* (National Oceanic and Atmospheric Administration, 2018), pp. 49–52.

20. K. Bi, L. Xie, H. Zhang, X. Chen, X. Gu, Q. Tian, Accurate medium-range global weather forecasting with 3D neural networks. *Nature* **619**, 533–538 (2023).

21. R. Lam, A. Sanchez-Gonzalez, M. Willson, P. Wirnsberger, M. Fortunato, F. Alet, S. Ravuri, T. Ewalds, Z. Eaton-Rosen, W. Hu, A. Merose, S. Hoyer, G. Holland, O. Vinyals, J. Stott, A. Pritzel, S. Mohamed, P. Battaglia, Learning skillful medium-range global weather forecasting. *Science* **382**, 1416–1421 (2023).

22. I. Price, A. Sanchez-Gonzalez, F. Alet, T. R. Andersson, A. El-Kadi, D. Masters, T. Ewalds, J. Stott, S. Mohamed, P. Battaglia, R. Lam, M. Willson, Probabilistic weather forecasting with machine learning. *Nature* **637**, 84–90 (2025).

23. D. Kochkov, J. Yuval, I. Langmore, P. Norgaard, J. Smith, G. Mooers, M. Klöwer, J. Lottes, S. Rasp, P. Düben, S. Hatfield, P. Battaglia, A. Sanchez-Gonzalez, M. Willson, M. P. Brenner, S. Hoyer, Neural general circulation models for weather and climate. *Nature* **632**, 1060–1066 (2024).

24. Y.-G. Ham, J.-H. Kim, J.-J. Luo, Deep learning for multi-year ENSO forecasts. *Nature* **573**, 568–572 (2019).

25. F. Ling, J.-J. Luo, Y. Li, T. Tang, L. Bai, W. Ouyang, T. Yamagata, Multi-task machine learning improves multi-seasonal prediction of the Indian Ocean Dipole. *Nat. Commun.* **13**, 7681 (2022).

26. A. Subel, L. Zanna, Building ocean climate emulators. arXiv:2402.04342 [physics.ao-ph] (2024).

27. A. Vaswani, N. Shazeer, N. Parmar, J. Uszkoreit, L. Jones, A. N. Gomez, Ł. Kaiser, I. Polosukhin, Attention is all you need. *Adv. Neural Inf. Process. Syst.* **30**, 5998–6008 (2017).

28. V. Eyring, S. Bony, G. A. Meehl, C. A. Senior, B. Stevens, R. J. Stouffer, K. E. Taylor, Overview of the coupled model intercomparison project phase 6 (CMIP6) experimental design and organization. *Geosci. Model Dev.* **9**, 1937–1958 (2016).

29. D. W. Behringer, M. Ji, A. Leetmaa, An improved coupled model for ENSO prediction and implications for ocean initialization. Part I: The ocean data assimilation system. *Mon. Weather Rev.* **126**, 1013–1021 (1998).

30. F. J. Doblas-Reyes, J. García-Serrano, F. Lienert, A. P. Biescas, L. R. L. Rodrigues, Seasonal climate predictability and forecasting: Status and prospects. *Wiley Interdiscip. Rev. Clim. Change* **4**, 245–268 (2013).

31. L. Magnusson, M. Alonso-Balmaseda, S. Corti, F. Molteni, T. Stockdale, Evaluation of forecast strategies for seasonal and decadal forecasts in presence of systematic model errors. *Clim. Dyn.* **41**, 2393–2409 (2013).

32. S. Huihang, W. Yiguo, L. Jingjia, Impact of ocean data assimilation on initial conditions and skills of seasonal-to-interannual climate prediction. *J. Trop. Oceanogr.* **41**, 75–90 (2022).

33. B. P. Kirtman, D. Min, J. M. Infanti, J. L. Kinter, D. A. Paolino, Q. Zhang, H. van den Dool, S. Saha, M. P. Mendez, E. Becker, P. Peng, P. Tripp, J. Huang, D. G. DeWitt, M. K. Tippett, A. G. Barnston, S. Li, A. Rosati, S. D. Schubert, M. Rienecker, M. Suarez, Z. E. Li, J. Marshak, Y.-K. Lim, J. Tribbia, K. Pegion, W. J. Merryfield, B. Denis, E. F. Wood, The North American multimodel ensemble: Phase-1 seasonal-to-interannual prediction; Phase-2 toward developing intraseasonal prediction. *Bull. Am. Meteorol. Soc.* **95**, 585–601 (2014).

34. J.-J. Luo, S. Masson, S. Behera, S. Shingu, T. Yamagata, Seasonal climate predictability in a coupled OAGCM using a different approach for ensemble forecasts. *J. Clim.* **18**, 4474–4497 (2005).

35. Z. Chen, V. Badrinarayanan, C.-Y. Lee, A. Rabinovich, “GradNorm: Gradient normalization for adaptive loss balancing in deep multitask networks,” in *Proceedings of the 35th International Conference on Machine Learning (ICML)* (PMLR, 2018), pp. 794–803.

36. J. Bjerknes, Atmospheric teleconnections from the equatorial Pacific. *Mon. Weather Rev.* **97**, 163–172 (1969).

37. A. Timmermann, S.-I. An, J.-S. Kug, F.-F. Jin, W. Cai, A. Capotondi, K. M. Cobb, M. Lengaigne, M. J. McPhaden, M. F. Stuecker, K. Stein, A. T. Wittenberg, K.-S. Yun, T. Bayr, H.-C. Chen, Y. Chikamoto, B. Dewitte, D. Dommenget, P. Grothe, E. Guilyardi, Y.-G. Ham, M. Hayashi, S. Ineson, D. Kang, S. Kim, W. Kim, J.-Y. Lee, T. Li, J.-J. Luo, S. McGregor, Y. Planton, S. Power, H. Rashid, H.-L. Ren, A. Santoso, K. Takahashi, A. Todd, G. Wang, G. Wang, R. Xie, W.-H. Yang, S.-W. Yeh, J. Yoon, E. Zeller, X. Zhang, El Niño–Southern Oscillation complexity. *Nature* **559**, 535–545 (2018).

38. P. Lyu, T. Tang, F. Ling, J.-J. Luo, N. Boers, W. Ouyang, L. Bai, ResoNet: Robust and explainable ENSO forecasts with hybrid convolution and transformer networks. *Adv. Atmos. Sci.* **41**, 1289–1298 (2024).

39. K. E. Trenberth, The definition of El Niño. *Bull. Am. Meteorol. Soc.* **78**, 2771–2778 (1997).

40. M. A. Cane, The evolution of El Niño, past and future. *Earth Planet. Sci. Lett.* **230**, 227–240 (2005).

41. L. Zhou, R.-H. Zhang, A self-attention–based neural network for three-dimensional multivariate modeling and its skillful ENSO predictions. *Sci. Adv.* **9**, eadf2827 (2023).

42. T. Izumo, M. Colin, F.-F. Jin, B. Pagli, The hybrid recharge delayed oscillator: A more realistic El Niño conceptual model. *J. Clim.* **37**, 2765–2787 (2024).

43. T. P. Guilderson, D. P. Schrag, Abrupt shift in subsurface temperatures in the tropical Pacific associated with changes in El Niño. *Science* **281**, 240–243 (1998).

44. K. Ashok, S. K. Behera, S. A. Rao, H. Weng, T. Yamagata, El Niño Modoki and its possible teleconnection. *J. Geophys. Res. Oceans* **112**, C11007 (2007).

45. K. Takahashi, A. Montecinos, K. Goubanova, B. Dewitte, ENSO regimes: Reinterpreting the canonical and Modoki El Niño. *Geophys. Res. Lett.* **38**, L10704 (2011).

46. A. Capotondi, A. T. Wittenberg, M. Newman, E. D. Lorenzo, J.-Y. Yu, P. Braconnot, J. Cole, B. Dewitte, B. Giese, E. Guilyardi, F.-F. Jin, K. Karnauskas, B. Kirtman, T. Lee, N. Schneider, Y. Xue, S.-W. Yeh, Understanding ENSO diversity. *Bull. Am. Meteorol. Soc.* **96**, 921–938 (2015).

47. A. Capotondi, R. R. Rodrigues, A. Sen Gupta, J. A. Benthuysen, C. Deser, T. L. Frölicher, N. S. Lovenduski, D. J. Amaya, N. Le Grix, T. Xu, J. Hermes, N. J. Holbrook, C. Martinez-Villalobos, S. Masina, M. K. Roxy, A. Schaeffer, R. W. Schlegel, K. E. Smith, C. Wang, A global overview of marine heatwaves in a changing climate. *Commun. Earth Environ.* **5**, 701 (2024).

48. K. E. Smith, M. T. Burrows, A. J. Hobday, A. S. Gupta, P. J. Moore, M. Thomsen, T. Wernberg, D. A. Smale, Socioeconomic impacts of marine heatwaves: Global issues and opportunities. *Science* **374**, eabj3593 (2021).

49. J. A. Santora, N. J. Mantua, I. D. Schroeder, J. C. Field, E. L. Hazen, S. J. Bograd, W. J. Sydeman, B. K. Wells, J. Calambokidis, L. Saez, D. Lawson, K. A. Forney, Habitat compression and ecosystem shifts as potential links between marine heatwave and record whale entanglements. *Nat. Commun.* **11**, 536 (2020).

50. M. Marin, M. Feng, N. L. Bindoff, H. E. Phillips, Local drivers of extreme upper ocean marine heatwaves assessed using a global ocean circulation model. *Front. Clim.* **4**, 788390 (2022).

51. E. C. J. Oliver, J. A. Benthuysen, S. Darmaraki, M. G. Donat, A. J. Hobday, N. J. Holbrook, R. W. Schlegel, A. Sen Gupta, Marine heatwaves. *Ann. Rev. Mar. Sci.* **13**, 313–342 (2021).

52. M. G. Jacox, M. A. Alexander, D. Amaya, E. Becker, S. J. Bograd, S. Brodie, E. L. Hazen, M. Pozo Buil, D. Tommasi, Global seasonal forecasts of marine heatwaves. *Nature* **604**, 486–490 (2022).

53. R. McAdam, S. Masina, S. Gualdi, Seasonal forecasting of subsurface marine heatwaves. *Commun. Earth Environ.* **4**, 225 (2023).

54. G. A. Meehl, L. Goddard, J. Murphy, R. J. Stouffer, G. Boer, G. Danabasoglu, K. Dixon, M. A. Giorgetta, A. M. Greene, E. Hawkins, G. Hegerl, D. Karoly, N. Keenlyside, M. Kimoto, B. Kirtman, A. Navarra, R. Pulwarty, D. Smith, D. Stammer, T. Stockdale, Decadal prediction: Can it be skillful? *Bull. Am. Meteorol. Soc.* **90**, 1467–1486 (2009).

55. G. J. Boer, D. M. Smith, C. Cassou, F. Doblas-Reyes, G. Danabasoglu, B. Kirtman, Y. Kushnir, M. Kimoto, G. A. Meehl, R. Msadek, W. A. Mueller, K. E. Taylor, F. Zwiers, M. Rixen, Y. Ruprich-Robert, R. Eade, The Decadal Climate Prediction Project (DCPP) contribution to CMIP6. *Geosci. Model Dev.* **9**, 3751–3777 (2016).

56. S. Zhao, F.-F. Jin, M. F. Stuecker, P. R. Thompson, J.-S. Kug, M. J. McPhaden, M. A. Cane, A. T. Wittenberg, W. Cai, Explainable El Niño predictability from climate mode interactions. *Nature* **630**, 891–898 (2024).

57. J. A. Carton, B. S. Giese, A reanalysis of ocean climate using Simple Ocean Data Assimilation (SODA). *Mon. Weather Rev.* **136**, 2999–3017 (2008).

58. Copernicus Climate Change Service, Climate Data Store, ORAS5 global ocean reanalysis monthly data from 1958 to present, Copernicus Climate Change Service (C3S) Climate Data Store (CDS) (2021); https://doi.org/10.24381/cds.67e8eeb7.

59. I. Loshchilov, F. Hutter, Decoupled weight decay regularization. arXiv:1711.05101 [cs.LG] (2019).

60. A. Dosovitskiy, L. Beyer, A. Kolesnikov, D. Weissenborn, X. Zhai, T. Unterthiner, M. Dehghani, M. Minderer, G. Heigold, S. Gelly, An image is worth 16x16 words: Transformers for image recognition at scale. arXiv:2010.11929 [cs.CV] (2021).

61. Z. Liu, Y. Lin, Y. Cao, H. Hu, Y. Wei, Z. Zhang, S. Lin, B. Guo, "Swin transformer: Hierarchical vision transformer using shifted windows," in *Proceedings of the IEEE Conference on Computer Vision and Pattern Recognition (CVPR)* (IEEE, 2021), pp. 10012–10022.

62. J. Su, M. Ahmed, Y. Lu, S. Pan, W. Bo, Y. Liu, Roformer: Enhanced transformer with rotary position embedding. *Neurocomputing* **568**, 127063 (2024).

63. X. Wang, R. Wang, N. Hu, P. Wang, P. Huo, G. Wang, H. Wang, S. Wang, J. Zhu, J. Xu, Xihe: A data-driven model for global ocean eddy-resolving forecasting. arXiv:2402.02995 [physics.ao-ph] (2024).

64. N. Shazeer, A. Mirhoseini, K. Maziarz, A. Davis, Q. Le, G. Hinton, J. Dean, Outrageously large neural networks: The sparsely-gated mixture-of-experts layer. arXiv:1701.06538 (2017).

65. J. L. Ba, J. R. Kiros, G. E. Hinton, Layer normalization. arXiv:1607.06450 [cs.LG] (2016).

66. C. A. Ferro, D. B. Stephenson, Extremal dependence indices: Improved verification measures for deterministic forecasts of rare binary events. *Weather Forecast.* **26**, 699–713 (2011).

67. A. J. Hobday, L. V. Alexander, S. E. Perkins, D. A. Smale, S. C. Straub, E. C. J. Oliver, J. A. Benthuysen, M. T. Burrows, M. G. Donat, M. Feng, N. J. Holbrook, P. J. Moore, H. A. Scannell, A. Sen Gupta, T. Wernberg, A hierarchical approach to defining marine heatwaves. *Prog. Oceanogr.* **141**, 227–238 (2016).

68. M. G. Jacox, M. A. Alexander, S. J. Bograd, J. D. Scott, Thermal displacement by marine heatwaves. *Nature* **584**, 82–86 (2020).

69. B. J. Henley, J. Gergis, D. J. Karoly, S. Power, J. Kennedy, C. K. Folland, A tripole index for the interdecadal Pacific oscillation. *Clim. Dyn.* **45**, 3077–3090 (2015).

70. H. Liu, Z. Song, X. Wang, V. Misra, An ocean perspective on CMIP6 climate model evaluations. *Deep Sea Res. 2 Top Stud. Oceanogr*. **201**, 105120, (2022).

71. H. Liao, C. Wang, Z. Song, ENSO phase-locking biases from the CMIP5 to CMIP6 models and a possible explanation. *Deep Sea Res. 2 Top Stud. Oceanogr*. **189**, 104943, (2021).

72. Z. Qiu, F. Qiao, C. J. Jang, L. Zhang, Z. Song, Evaluation and projection of global marine heatwaves based on CMIP6 models. *Deep Sea Res. 2 Top Stud. Oceanogr*. **194**, 104998 (2021).

73. N. Saji, B. N. Goswami, P. Vinayachandran, T. Yamagata, A dipole mode in the tropical Indian Ocean. *Nature* **401**, 360–363 (1999).

74. S. E. Zebiak, Air–sea interaction in the equatorial Atlantic region. *J. Clim.* **6**, 1567–1586 (1993).

75. P. Chang, L. Ji, H. Li, A decadal climate variation in the tropical Atlantic Ocean from thermodynamic air-sea interactions. *Nature* **385**, 516–518 (1997).
